# Microphysics of Time-Dependent Deformation of Clay-Bearing Sandstone: Grain Shape Matters

**DOI:** 10.1007/s00603-025-05192-2

**Published:** 2026-01-13

**Authors:** Takahiro Shinohara, Christopher J. Spiers, Suzanne J. T. Hangx

**Affiliations:** 1 Department of Earth Sciences, Utrecht University, Princetonlaan 4, 3584CB Utrecht, The Netherlands; 2 Present Address: Department of Geoscience and Engineering, Delft University of Technology, Stevinweg 1, 2628CN Delft, The Netherlands

**Keywords:** Stress corrosion cracking, Frictional grain boundary sliding, Reservoir compaction, Grain aspect ratio, Deformation mechanism, Physics-based model

## Abstract

Reliable assessment of the long-term effects of hydrocarbon production from sandstone reservoirs, on induced subsidence and seismicity, requires an understanding of the processes that lead to associated reservoir compaction. The compaction is typically small (strain < 1%) and often considered to be purely elastic, but in reality is partly permanent and possibly even time-dependent. This means compaction may continue even after production stops. Empirical models are often used to evaluate future reservoir compaction, however the reliability of such extrapolation in time is questionable without underpinning in terms of the governing processes. We develop a simplified microphysical model with a variable microstructure (i.e., unit cells with varying grain packings), aimed at capturing the deformation mechanisms observed in triaxial compression experiments on clay-bearing Bleurswiller sandstone (porosity 21%). The mechanisms include rate-independent consolidation of intergranular clay films, rate-dependent intergranular slip along the clay films and stress corrosion cracking of quartz/clastic grains. Our model predicts mechanical behaviour consistent with the main features and trends observed in the experimental data. Stresses and strains are localized in unit cells with relatively low and high grain contact inclination, respectively, over the full range of deviatoric loading. Sensitivity analysis showed that quartz/clastic grain shape (aspect ratio) is an important factor determining the modelled mechanical behaviour. The present model provides mechanistic underpinning for existing geomechanical models accounting for rate-dependent deformation relevant to hydrocarbon production-induced subsidence and seismicity, at field conditions and timescales.

## Highlights

- Microphysical model explains mechanical behaviour of clay-bearing, porous sandstone, including time-dependent inelastic behaviour
- A variable model microstructure causes a range of grain boundary sliding rates, leading to stress and strain heterogeneity
- Grain aspect ratio is a key factor determining inelastic mechanical behaviour

## Introduction

Understanding the physical and chemical processes operating in a reservoir system is key to reliably assessing the long-term effects of hydrocarbon production from sandstone reservoirs on induced subsidence and seismicity (Mallman and Zoback 2007; Van Thienen-Visser and Breunese 2015; Van Wees et al. 2018). Subsidence and seismicity are generally caused by reservoir compaction resulting from pore pressure reduction and/or thermal contraction due to cooling. Reservoir compaction is often quantified purely with linear, reversible and rate-insensitive poroelasticity theory (Bourne and Oates 2020; Dempsey and Suckale 2017; Laurent et al. 1993; Segall 1992; Van Eijs et al. 2006). However, a significant part of the compaction, even at small strains of < 0.5%, is suggested to be permanent (Santarelli et al. 1998; Shalev et al. 2014) and possibly time-dependent (Bernabé et al. 1994; Hol et al. 2015, 2018; Pijnenburg et al. 2018). Even though typical reservoir strains are small, differential compaction and associated gradients in deformation across pre-existing faults may increase shear tractions and/or decrease normal stresses to trigger seismogenic rupture (Bourne et al. 2014; Buijze et al. 2017; Jansen et al. 2019; Mulders 2003). Constraining the magnitude of the in situ reservoir strain and particularly of the inelastic contribution, including the time-dependent component, is key for realistic geomechanical modelling of induced subsidence and seismicity (Buijze et al. 2017; Candela et al. 2019; J. Smith et al. 2019; Van Thienen-Visser and Breunese 2015; Van Wees et al. 2018). To date, however, much research on sandstone deformation has focused on inelastic deformation occurring at large strains (1–15%) (e.g., Baud et al. 2004; Cartwright-Taylor et al. 2022; Fortin et al. 2006; Tembe et al. 2007; Wong et al. 1997).

Recent studies, performed at typical laboratory strain rates (${10}^{-5} {\mathrm{s}}^{-1}$) and at strains relevant to depleting sandstone reservoirs (< 1%), suggest that time- or rate-insensitive compaction of and slip along intergranular clay films primarily govern inelastic deformation of sandstones containing intergranular clay films (Pijnenburg et al. 2019a, 2019b; Verberne et al. 2020). In other sandstones with little intergranular clay films, grain rearrangement is the dominant inelastic deformation mechanism (Shalev et al. 2014). However, experiments at strain rates down to ${10}^{-9} {\mathrm{s}}^{-1}$ have revealed that slip along intergranular clay films shows a significant rate-dependence, leading to time- or rate-dependent inelastic deformation (Pijnenburg et al. 2018; Shinohara et al. 2024; Shinohara et al. 2025b). Stress corrosion cracking has also been shown to be a crucial time- or rate-dependent deformation mechanism in sandstones, occurring at strains of 0.5%–1% and strain rates down to ${10}^{-9} {\mathrm{s}}^{-1}$ (Brantut et al. 2014; Cartwright-Taylor et al. 2022; Heap et al. 2015; Pijnenburg et al. 2018; Shinohara et al. 2024; Shinohara et al. 2025b; Stanchits et al. 2009). However, extrapolation of such laboratory-derived constraints on reservoir compaction behaviour, to field timescales and conditions, requires underpinning by a much better understanding of the governing processes and associated constitutive response (Spiers et al. 2017).

This is particularly interesting in Europe’s largest gas field, the densely populated Groningen gas field in the Netherlands, where all production from the Slochteren reservoir was halted on 19 April 2024 (Ministry of Economic Affairs and Climate Policy 2024) in an attempt to halt the widespread induced seismicity and surface subsidence that have been observed over the last several decades. However, it is unclear how much, and for how long, time-dependent compaction and associated seismicity will occur now that production has stopped. For this field, a number of empirical rate/time-dependent compaction models (De Waal and Smits 1988; Mossop 2012; Pruiksma et al. 2015) have been calibrated to subsidence data and used to provide history-matched estimates of future subsidence (NAM 2021; Van Thienen-Visser and Breunese 2015). However, a mechanistic basis for those empirical models and the underlying experimental data is still lacking, which hampers reliable extrapolation of the model results to the decade timescales relevant to post-abandonment behaviour.

Mechanistic bases for sandstone deformation behaviour are increasingly underpinned using physics-based models (Einav 2007a, 2007b; Guéguen and Fortin 2013; Pijnenburg and Spiers 2020). The role played by time-independent intragranular cracking has been investigated through the use of criteria for the onset of pervasive, time-independent, intragranular cracking (Guéguen and Fortin 2013; Wong et al. 1997; J. Zhang et al. 1990), or through thermodynamically based breakage mechanics models that determine the stress–strain behaviour (Einav 2007a, 2007b; Tengattini et al. 2014). The thermodynamically based approach has also been extended to account for rate-dependent grain breakage caused by stress corrosion cracking (Y. Zhang and Buscarnera 2017). However, in most cases the focus lies on describing high strain behaviour (1–15% strain) far beyond the stress and strain conditions of depleting sandstone reservoirs. For depletion-relevant strains, the role played by rate-insensitive compaction and shear of intergranular clay films in determining the stress–strain behaviour (Pijnenburg et al. 2019a) has been addressed via a microphysical model (Pijnenburg and Spiers 2020). To date, however, the applicability of this model remains questionable for reservoir compaction behaviour after production stops, since time- or rate-dependent deformation is neglected.

In this study, we develop and test a series of microphysical models addressing the inelastic deformation processes, including rate-dependent processes, observed in the conventional triaxial compression experiments conducted on clay-bearing Bleurswiller sandstone (Shinohara et al. 2024), at an effective confining pressure of 29 MPa and a temperature of 100 ℃. The Bleurswiller sandstone has petrophysical and mechanical properties comparable to the Slochteren sandstone comprising the reservoir of the Groningen gas field. First, we (re)evaluate whether rate-dependent intergranular clay slip/shear and consolidation can quantitatively account for the inelastic behaviour observed at all stress levels investigated. The role of stress corrosion cracking of quartz/clastic grains in determining the peak stress observed in the experiments is also investigated.

The simplified models obtained aim to test whether the microphysical mechanisms described in Shinohara et al. (2024) can (semi-)quantitatively account for the rate-dependent inelastic behaviour of porous, clay-bearing Bleurswiller sandstone observed at the sample scale (1–10 cm) in our triaxial compression experiments. If that is the case, the underlying physical processes and deformation behaviour will be explored in further detail in future, using more rigorous approaches for linking grain-scale to aggregate-scale (> 10 cm) behaviour, i.e., such as homogenization treatments (Fortin et al. 2003), granular mechanics modelling (Einav 2007a, 2007b; Tengattini et al. 2014) and/or the discrete element method (Kawamoto et al. 2016, 2018).

## Decoupling of Elastic and Inelastic Deformation

We adopt the convention that compressive stresses and strains are positive. All stresses (denoted σ) supported by the sandstone framework are understood to be Terzaghi effective values, i.e., total stress ($\sigma {\prime}$) minus pore pressure (${P}_{\mathrm{p}}$). A list of the symbols used is given in Table 1.

**Table 1:** Explanation of the symbols used

a	Meridional crack length (m)	${v}_{\mathrm{slip}0}$	Reference grain boundary slip velocity (m/s)
${\widetilde{a}}_{\mu }$	Rate-dependent coefficient in RSF terminology (–)	W	Mechanical work supplied (J)
B	Constant for the consolidation behaviour of illite (–)	$d{W}_{\mathrm{ext}}$	External work increment per unit volume by splitting ($\mathrm{J}/{\mathrm{m}}^{3}$)
b	Crack width (m)	$d{W}_{\mathrm{int}}$	Internal work increment per unit volume by splitting ($\mathrm{J}/{\mathrm{m}}^{3}$)
c	Half crack opening upon meridional splitting (m)	z	Initial intergranular clay film thickness (m)
d	Asperity and initial flaw spacing at grain contact (m)	${z}^{*}$	Grain contact clay film thickness upon splitting (m)
E	Young’s modulus (Pa)	α	Angle between grain contact and horizontal axis (rad)
${E}_{\mathrm{g}}$	Grain Young’s modulus (Pa)	${\gamma }_{\mathrm{env}}$	Specific interfacial energy of fluid-filled cracks within grains ($\mathrm{J}/{\mathrm{m}}^{2}$)
${F}_{B}$	Axial grain contact force (N)	Δ	Energy release rate (J/s)
${f}_{\mathrm{el}}$	Elastic deformation energy stored in a grain (J)	$\widetilde{\varepsilon }$	Grain contact normal strain on A or B (–)
${f}_{\mathrm{el}-\mathrm{b}}$	Elastic energy stored in a grain due to bending (J)	${\varepsilon }_{i}^{cr}$	Total strain due to intragranular crackings in direction i=1,2,3 (–)
${f}_{\mathrm{el}-\mathrm{c}}$	Elastic energy stored in a grain due to compression (J)	${\varepsilon }_{i}^{cr,m}$	Strain due to multi-edge cracking in direction i=1,2,3 (–)
${f}_{s}$	Crack surface energy per grain (J)	${\varepsilon }_{i}^{cr,s}$	Strain due to intragranular splitting in direction i=1,2,3 (–)
G	Mechanical energy release rate per unit crack extension (N/m)	${\varepsilon }_{i}^{\mathrm{el}}$	Elastic strain in direction i=1,2,3 (–)
g	Net crack extension force per unit crack width (N/m)	${\varepsilon }_{i}^{\mathrm{inel}}$	Inelastic strain in direction i=1,2,3 (–)
I	Moment of inertia about a meridional crack (Nm)	${\varepsilon }_{i}^{\mathrm{tot}}$	Total strain in direction i=1,2,3 (–)
k	Grain coordination number (–)	θ	Angle between grain long axis ${R}_{\mathrm{b}}$ and horizontal axis (deg)
l	Bending axis of the meridional crack (m)	$\kappa , \lambda$	Geometrical constants describing the meridional cracking (–)
M	Bending moment about a meridional crack (Nm)	$\widetilde{\mu }$	Grain contact friction coefficient (–)
m	Grain contact proportion not in contact with clay film at the open crack end (–)	${\widetilde{\mu }}_{0}$	Reference grain contact friction coefficient (–)
n	Grain contact proportion in contact with clay film at closed crack end (–)	ν	Poisson’s ratio (Pa)
P	Mean effective stress $\frac{{\sigma }_{1}+{\sigma }_{2}+{\sigma }_{3}}{3}-{P}_{p}$ (Pa)	$\widetilde{\sigma }$	Grain contact normal stress on A or B (Pa)
${P}_{\mathrm{c}}$	Confining pressure (Pa)	${\sigma }_{i}$	Principal effective stresses (i=1,2,3) (Pa)
${P}_{\mathrm{p}}$	Pore pressure (Pa)	$\sigma^{\prime}_{i}$	Principal total stresses (i=1,2,3) (Pa)
Q	Differential stress (${\sigma }_{1}-{\sigma }_{3}$) (Pa)	${\sigma }_{1}^{*}$	Axial stress upon splitting (Pa)
${R}_{\mathrm{a}}$	Grain radius along short axis of ellipsoidal grain (m)	${\widetilde{\sigma }}_{B}^{*}$	Grain contact normal stress on B upon splitting (Pa)
${R}_{\mathrm{b}}$	Grain radius along long axis of ellipsoidal grain (m)	$\widetilde{\tau }$	Grain contact shear stress on A or B (Pa)
${R}_{\mathrm{env}}$	Crack surface energy (= ${2\gamma }_{\mathrm{env}}$) ($\mathrm{J}/{\mathrm{m}}^{2}$)	χ	Aspect ratio of ellipsoidal grain ($={R}_{\mathrm{a}}/{R}_{\mathrm{b}}$) (–)
${R}_{\mathrm{s}}$	Radius of equivalent spherical grain (m)	ψ	Equivalent grain contact angle (rad)
${R}_{\mathrm{sc}}$	Equivalent spherical grain radius towards grain contact (m)	${\phi }_{0}$	Initial porosity (–)
r	Model grain contact radius (m)	${\phi }_{\mathrm{c}}$	Critical porosity (–)
${s}_{i}$	Internal entropy (J/K)	${\phi }_{\mathrm{cr}}$	Intragranular porosity fraction assumed to prevail after cracking (–)
T	Temperature (℃)	$\Delta {\phi }_{\mathrm{inel}}$	Inelastic porosity reduction (–)
${v}_{\mathrm{slip}}$	Grain boundary slip velocity (m/s)	$\Delta {\phi }_{\mathrm{tot}}$	Total porosity reduction (–)

In general, the principal total strain rates (${\dot{\varepsilon }}_{i}^{\mathrm{tot}}$, i=1,2 or 3) are given as the sum of the elastic (${\dot{\varepsilon }}_{i}^{\mathrm{el}}$) and inelastic (${\dot{\varepsilon }}_{i}^{\mathrm{inel}}$) components, as:

1 $$\begin{array}{*{20}c} {\begin{array}{*{20}c} {\dot{\varepsilon }_{i}^{{{\mathrm{tot}}}} = \dot{\varepsilon }_{i}^{{{\mathrm{el}}}} + \dot{\varepsilon }_{i}^{{{\mathrm{inel}}}} } \\ \end{array} } \\ \end{array} ,$$

where ${\dot{\varepsilon }}_{i}^{\mathrm{inel}}$ includes both time-independent and time-dependent inelastic strain contributions. In using Eq. 1, we assume that the elastic and the inelastic contributions are fully decoupled, so that each can be evaluated independently of the other.

## Quantifying the Elastic Contribution

The elastic strain contribution is quantified here using constant elastic properties, following the observation that mechanical data on Bleurswiller sandstone show near-linear elastic behaviour, i.e., near-constant elastic properties, at least up to a differential stress of $60 \mathrm{MPa}$ at $T=100^\circ{\rm C}$, ${P}_{\mathrm{c}}=39 \mathrm{MPa}$ and ${P}_{\mathrm{p}}=10 \mathrm{MPa}$ (Shinohara et al. 2024). By fitting linear elastic relationships to the mechanical data in differential stress Q vs. axial strain ${\varepsilon }_{1}^{\mathrm{tot}}$ and in mean effective stress P vs. total porosity reduction $\Delta {\phi }_{\mathrm{tot}}$ spaces, we obtained values for Young’s modulus E and Poisson’s ratio ν of $13.7\text{ GPa}$ and 0.28, respectively (Table 2). We use these elastic values for all differential stress levels, assuming this linearity holds at differential stress above 60 MPa.

**Table 2:** Input parameters used to construct the plots shown in Figs. 5 and 6

Parameter	Description	Values	Data source
Grain geometrical parameters			
${\phi }_{0}$ (%)	Initial porosity of our Bleurswiller sandstone	22.1	Shinohara et al. (2024)
${ R}_{\mathrm{b}}$ (μm)	Grain radius along long axis	110	Value obtained from microstructural analysis
${ R}_{\mathrm{a}}/{R}_{\mathrm{b}}$ (= χ)	Grain aspect ratio	0.58	Fig. 1d
Elastic parameters			
E (GPa)	Young’s modulus of our Bleurswiller sandstone	13.7	Experimentally obtained (Shinohara et al. 2024)
ν (–)	Poisson’s ratio of our Bleurswiller sandstone	0.28	Experimentally obtained (Shinohara et al. 2024)
Intergranular clay parameters			
z (μm)	Characteristic initial clay film thickness	2.5	Assumed here based on microstructure (e.g., Fig. 1f)
B (–)	Constant describing the consolidation behaviour of illite (cf. Eq. 9)	0.05	Pijnenburg and Spiers (2020)
${\widetilde{ \mu }}_{0}$ (–)	Friction coefficient of the intergranular illite at a reference velocity ${v}_{\mathrm{slip}0}$	0.35	Shinohara et al. (2024), Tembe et al. (2010)
${ v}_{\mathrm{slip}0}$ (m/s)	Reference velocity	${10}^{-6}$	Experimentally applied value (Hunfeld et al. 2017)
${\widetilde{ a}}_{\mu }$ (–)	Rate-dependent coefficient constituting a direct effect in RSF terminology	0.005	Experimentally obtained (Hunfeld et al. 2017)
Stress corrosion cracking parameters			
${\gamma }_{\mathrm{env}}$ ($\mathrm{J}/{\mathrm{m}}^{2}$)	Specific interfacial energy of fluid-filled cracks	1.46	Value for quartz (Du and De Leeuw 2006)
${ v}_{\mathrm{cr}0}$ (m/s)	Constant describing the subcritical crack velocity (cf. Eq. 30)	${10}^{-8.1}$	Value for quartz at $80 ^\circ{\rm C}$ (Darot and Guéguen 1986)
β (–)	Constant describing the subcritical crack velocity (cf. Eq. 30)	5.9	Value for quartz at $80 ^\circ{\rm C}$ (Darot and Guéguen 1986)
${ E}_{\mathrm{g}}$ (GPa)	Young’s modulus of sand grains	96	Value for quartz (e.g., J. Zhang et al. 1990)
λ (–)	Geometrical constant describing the meridional cracking (cf. Eq. 25)	0.5	Assumed here
κ (–)	Geometrical constant describing the meridional cracking (cf. Eq. 25)	0.5	Assumed here
${ \phi }_{\mathrm{cr}}$ (–)	Intragranular porosity fraction assumed to prevail after multi-edge cracking	0.4	Assumed here following Pijnenburg and Spiers (2020)

For present purposes (i.e., triaxial conditions at an effective confining pressure of $29 \, \mathrm{MPa}$, with ${\sigma }_{1}>{\sigma }_{2}={\sigma }_{3}={P}_{\mathrm{c}}$), the elastic strain contribution is quantified here as:

2a $$\begin{array}{c}{\dot{\varepsilon }}_{1}^{\mathrm{el}}=\frac{1}{E}{\dot{\sigma }}_{1,}\end{array}$$

2b $$\begin{array}{c}{\dot{\varepsilon }}_{\mathrm{2,3}}^{\mathrm{el}}=-\frac{\nu }{E}{\dot{\sigma }}_{1}. \end{array}$$

## Microstructural Model for Inelastic Deformation of Porous Sandstone

### Key Mechanical and Microstructural Observations

**Fig. 1:**
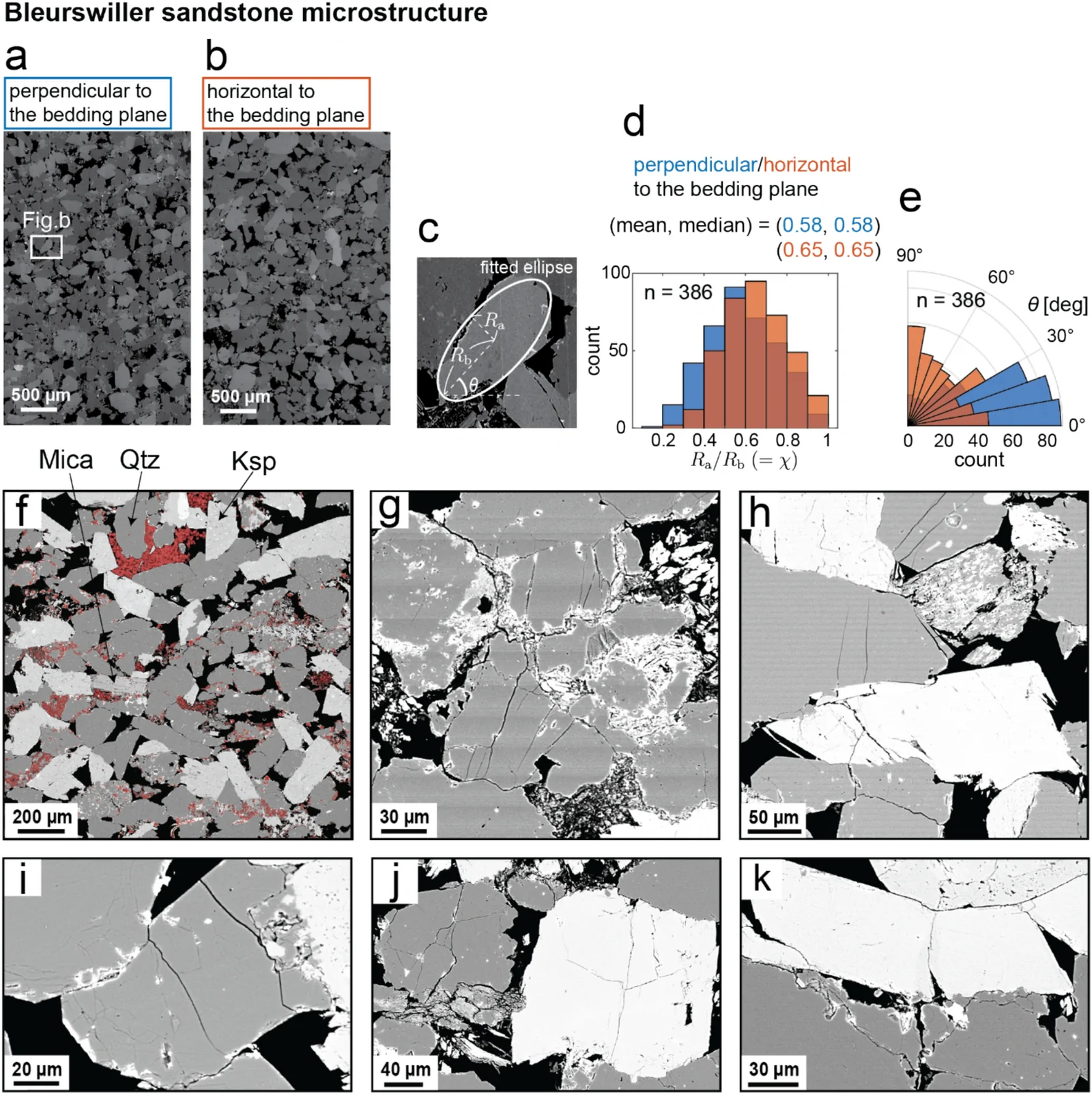
Key microstructural observations of **a–f** an undeformed Bleurswiller sandstone sample, and **g–k** a sample deformed up to peak differential stress at an axial strain rate of ${10}^{-6} \, {\mathrm{s}}^{-1}$, under ${P}_{\mathrm{eff}}=29$ MPa, $T=100 \,^{\circ}{\rm C}$. Overview image of an undeformed sample showing the presence of quartz, feldspar and mica, in a plane **a** perpendicular and **b** horizontal to bedding plane. **c** An example of an ellipse, with a short ${R}_{a}$ and a long ${R}_{b}$ axis, fitted to a framework grain to obtain a distribution of **d** aspect ratio of grains ${R}_{a}/{R}_{b}$ (=χ) and **e** angle θ between long axis and horizontal line, measured for 386 grains within undeformed samples, in a plane perpendicular (blue) and horizontal (orange) to the bedding plane. **f** Clay map superimposed onto a back scattered electron image (Shinohara et al. 2024). **g–k** Cracked quartz and feldspar grains observed within the deformed sample. Grains are cracked either by a single, meridional crack, or more pervasively cracked by multiple, roughly parallel cracks, referred to here as multi-edge cracks

We start model development by summarizing the key microstructural and mechanical features observed in Bleurswiller sandstone samples tested in Shinohara et al. (2024). The microstructural features are illustrated in Fig. 1, using scanning electron micrographs. Bleurswiller sandstone (Vosges, France) is a well-studied and homogeneous sandstone (Fig. 1a and b), with little sedimentary bedding. The sandstone has a porosity of $21.1\pm 0.5\%$ (Shinohara et al. 2024) and is composed of 66% quartz, 28% feldspar, 4% clay and 2% mica, and appears to be clay cemented (Heap et al. 2015). In a plane perpendicular to the bedding plane, the quartz and feldspar grains are blocky to elliptical (Fig. 1a and c), with an average aspect ratio (i.e., χ; ratio of short axis ${R}_{\mathrm{a}}$ to long axis ${R}_{\mathrm{b}}$) of 0.58 (blue histogram in Fig. 1d). The long axis is orientated near parallel to the bedding plane (blue histogram in Fig. 1e). On the other hand, in a plane parallel to the bedding plane, no preferential orientation of the long axis is observed (orange histogram in Fig. 1e), although the grains are also blocky to elliptical, with an average aspect ratio (χ=0.65; orange histogram in Fig. 1d) larger than that in the plane perpendicular to the bedding plane. The grains have an average grain diameter (equivalent spherical diameter ${R}_{\mathrm{s}}$) of 220 μm (Fortin et al. 2005). Grains appear randomly packed; hence, the grain contact planes (tangent planes) are distributed in orientation from horizontal to vertical (Fig. 1a and b).

The clay in the Bleurswiller sandstone mainly consists of kaolinite and illite and is present in the pore space and as thin films coating the pore walls and grain contacts (Fig. 1f; Shinohara et al. 2024). The thin clay films, present in some but not all grain contacts, typically have a thickness of up to several micrometres. Further quantification of the clay film thickness would be desirable, but is not attempted here, since any statistically meaningful analysis would require an elaborate microstructural study of a large number of grain contacts, which is not feasible in the present study.

**Fig. 2:**
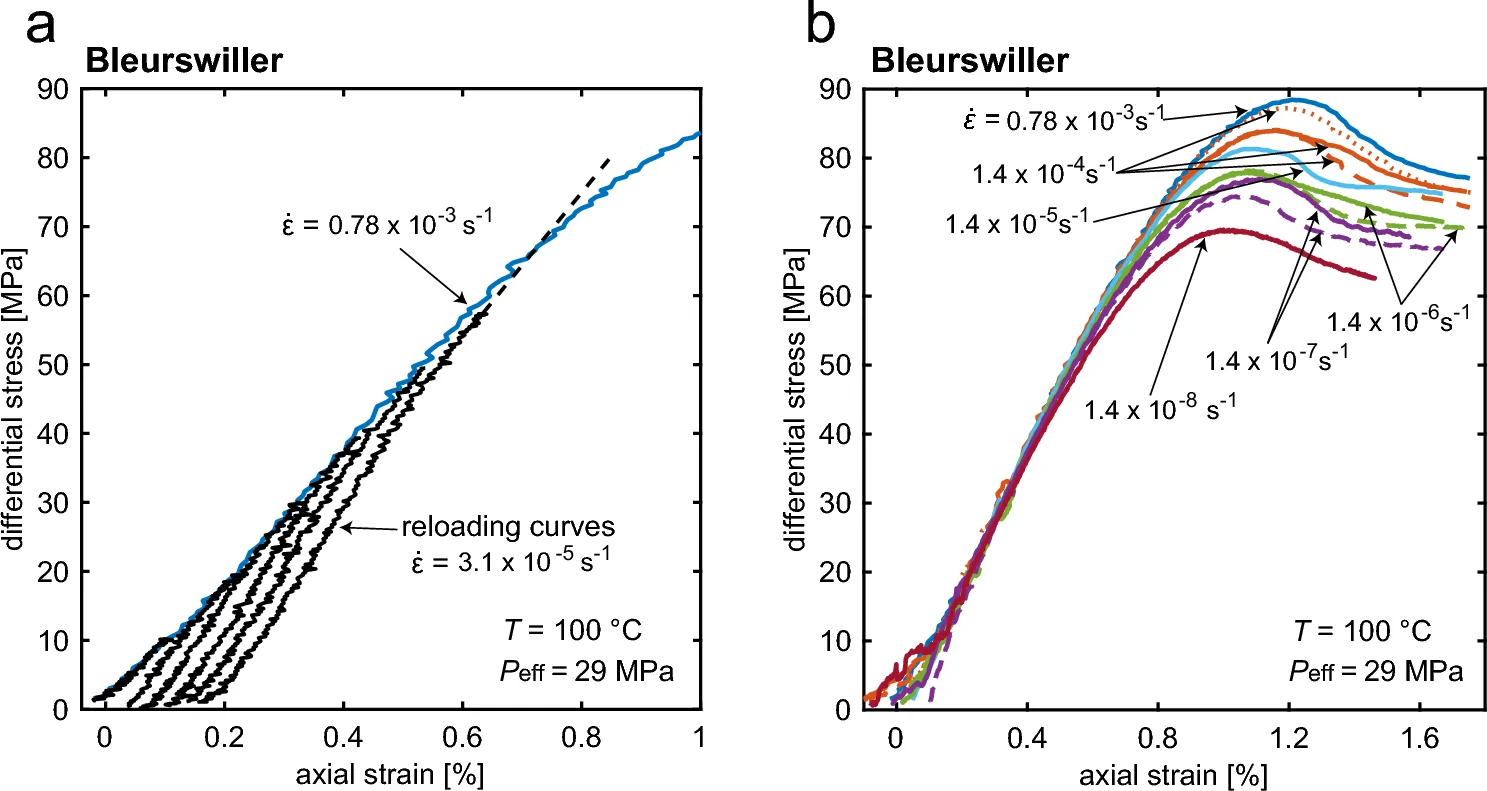
Plots showing differential stress versus axial strain curves for water-saturated Bleurswiller sandstone samples tested at a temperature of $100 \,^{\circ}{\rm C}$ and an effective confining pressure of 29 MPa: **a** for a cyclic loading experiment at an axial strain rate of ${10}^{-5} \,{\mathrm{s}}^{-1}$ (black curves), together with a constant strain rate experiment at $7.8\times {10}^{-4} \,{\mathrm{s}}^{-1}$ added for reference (blue curve), and **b** for constant strain rate experiments with strain rate varied per experiment, as indicated. Taken from Shinohara et al. (2024)

Bleurswiller sandstone samples, deformed at an axial strain rate of ${10}^{-5} \, {\mathrm{s}}^{-1}$, ${P}_{\mathrm{c}}=39 \, \mathrm{MPa}$, ${P}_{\mathrm{p}}=10 \, \mathrm{MPa}$ (effective confining pressure of $29 \,\mathrm{MPa}$) and $T=100 \,^{\circ}{\rm C}$, show combined elastic and inelastic strain at all differential stresses (up to 60 MPa) and total strains (0.1%–0.6%) investigated, i.e., over timescales of several minutes (Fig. 2a; Shinohara et al. 2024). Slochteren sandstone from the Groningen gas field, with petrophysical and mechanical properties comparable to our Bleurswiller sandstone, and tested at comparable stress, pressure and temperature conditions, over similar timescales, showed similar, combined elastic and inelastic deformation (Pijnenburg et al. 2019a), with the latter mostly due to compression (i.e., consolidation) of, and local slip on, the intergranular clay films (Pijnenburg et al. 2019b). Hence, we assume that clay consolidation and slip also play a role in inelastic strain development in our Bleurswiller sandstone.

The effect of axial strain rate on the Bleurswiller sandstone samples was systematically investigated (Shinohara et al. 2024), by extending the above triaxial tests down to strain rates of ${10}^{-8} \,{\mathrm{s}}^{-1}$. The results showed a systematic lowering of stress–strain curves with decreasing axial strain rate, at differential stresses exceeding 40–50% of peak stress (Fig. 2b). Further investigation of the deformation behaviour of Bleurswiller sandstone at varying confining pressure, temperature and pore fluid pH suggested that the observed rate effects are likely controlled by rate-dependent intergranular frictional sliding at lower differential stress (≤ 70% of peak strength), with an increased role of stress corrosion cracking at higher stress. This is in line with microstructural analysis, which showed that crack densities in samples deformed at axial strains of ${10}^{-4}$ and ${10}^{-8} \,{\mathrm{s}}^{-1}$, up to a differential stress of 60 MPa, were similar, suggesting that stress corrosion cracking played a negligible role at differential stresses below 60 MPa and strain rates above ${10}^{-8}{\text{ s}}^{-1}$ (Shinohara et al. 2024). In addition, samples deformed up to peak differential stress (75–80 MPa at ${10}^{-6} \,{\mathrm{s}}^{-1}$) revealed pervasive intragranular cracking, predominantly orientated near parallel to the axial compression direction (Fig. 1g and h). Those intragranular cracks often emanate from grain-to-grain contacts (Fig. 1g–k). Cracked grains are either split by a single, meridional crack (Fig. 1k), or else by multiple, roughly parallel cracks, with spacing down to several micrometres (Fig. 1g-j). The latter type will be referred to as multi-edge cracks. Cracks are typically open by 0.1–5 μm.

### Idealized Microstructure and Microstructural Unit Cell

**Fig. 3:**
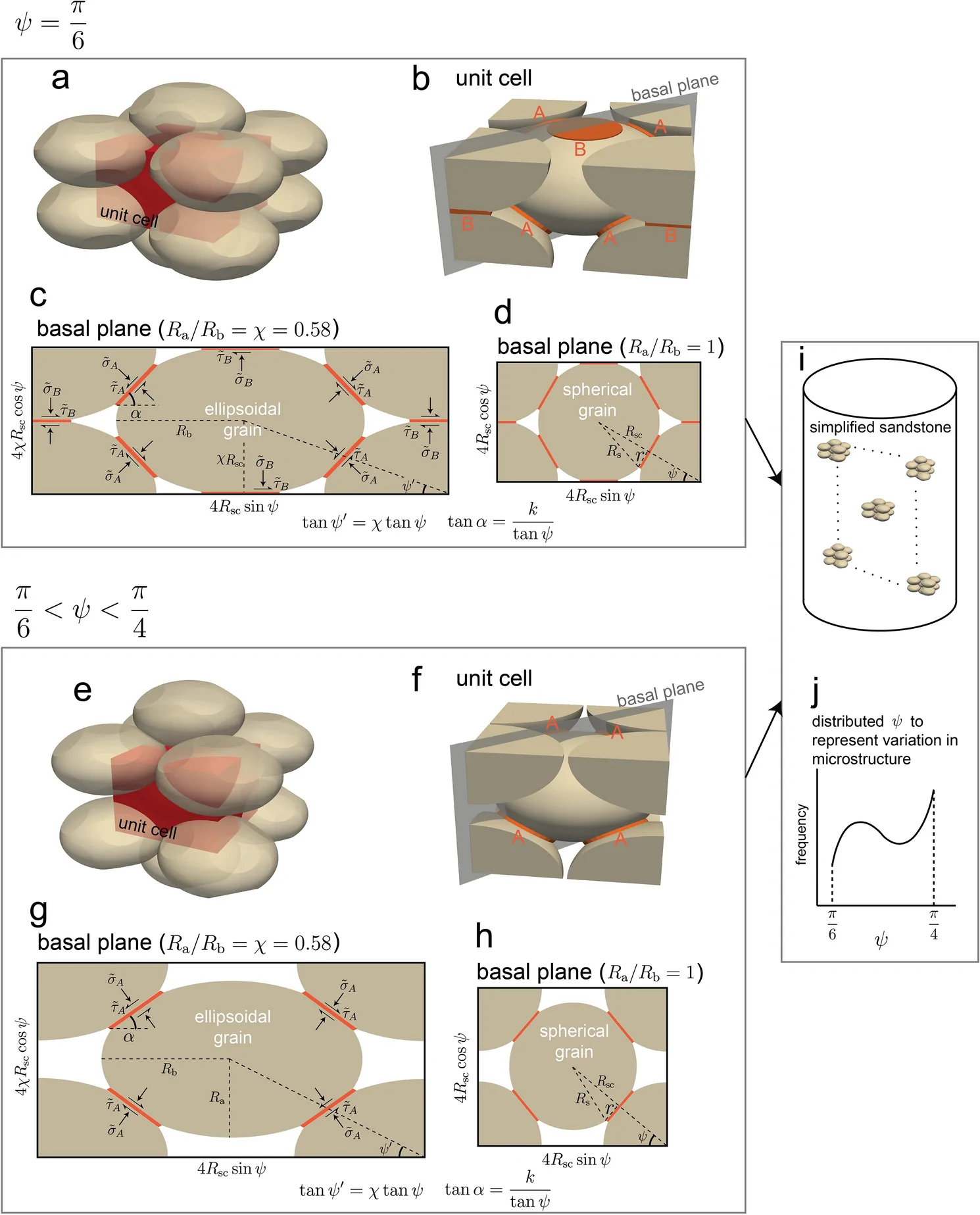
Assumed model microstructure for inelastic deformation of the clay-bearing Bleurswiller sandstone undergoing triaxial compression. The microstructure consists of simple packs of ellipsoidal quartz/clastic grains, characterized by a microstructural parameter **a–d** $\psi =\frac{\pi }{6}$ and **e–h** $\frac{\pi }{6}<\psi <\frac{\pi }{4}$*.* The grains have flattened contacts coated with clay films. **a, e** The two simple grain packs considered. **b, f)** Unit cell views of the simple grain packs. The basal planes are orientated so that they intersect the centre of each grain. Basal plane view of **c, g** the ellipsoidal grain packs and **d, h** equivalent spherical grains with a radius ${R}_{s}={R}_{b}$*.* **d, h** Grain geometrical parameters (grain radius towards grain contact ${R}_{sc}$ and grain contact radius r) are defined here. The **c, g** ellipsoidal grain packs were viewed as **d, h** spherical grain packs shrunk vertically by the aspect ratio of the ellipsoidal grains (χ)*.* Note that **a–d** the unit cell with $\psi =\frac{\pi }{6}$ contains inclined grain contacts A and horizontal grain contacts B, where normal $\widetilde{\sigma }$ and shear $\widetilde{\tau }$ stresses are applied due to axial ${\sigma }_{1}$ and lateral ${\sigma }_{2}$*,* ${\sigma }_{3}$ stresses externally imposed on the unit cells. By contrast, **e–h** the unit cell with $\frac{\pi }{6}<\psi <\frac{\pi }{4}$ only contains grain contact type A. **i** We assume that a simplified sandstone sample consists of the two unit cells with **j** distributed values of ψ to represent variation in microstructure

We assume an idealized and highly simplified model microstructure for Bleurswiller sandstone undergoing inelastic deformation, as shown in Fig. 3. This consists of a rectangular elementary volume or unit cell of sandstone containing a simple, 3D pack of quasi-ellipsoidal quartz grains (Fig. 3a and e) with flat, or truncated grain contacts, either circular (referred to as grain contacts B) or elliptical (referred to as grain contacts A), coated by clay films (Fig. 3b and f—coloured orange). The grains are radially symmetrical and oblate in the horizontal plane, with their horizontal long axes representing the sedimentary bedding orientation observed in the microstructure (Fig. 1e). Such radially symmetrical grain shape seems a representative, average grain shape, based on our microstructural observation that no preferential orientation of the grain long axis is observed in a plane horizontal to the bedding (i.e., radial) plane (Fig. 1e). To illustrate variable grain packing geometries observed in the microstructures, two idealized model microstructures are employed: one having an equivalent grain contact orientation defined by the angle $\psi =\frac{\pi }{6}$ (Fig. 3a–c; see Fig. 3d for the definition of ψ) and the other having $\frac{\pi }{6}<\psi <\frac{\pi }{4}$ (Fig. 3e–g; see Fig. 3h for the definition of ψ). Note that the unit cells with spherical grains (see Fig. 3d and h) are employed solely to define geometrical parameters and are not used for later model simulations. The grain radius along the long axis of grain ${R}_{\mathrm{b}}$ (= radius of equivalent spherical grain ${R}_{\mathrm{s}}$; cf. Fig. 3c and d and Fig. 3g and h) is taken to be 110 μm (Table 2). The grain contact radius (r) is approximated for a given initial porosity (${\phi }_{0}$) through:

3 $$\begin{array}{c}r=2{R}_{\mathrm{s}}\sqrt{\frac{2({\phi }_{\mathrm{c}}-{\phi }_{0})}{k}}, \end{array}$$

where ${\phi }_{\mathrm{c}}$ is the critical porosity equal to $1-\pi /12\mathrm{sin}\psi {\left(\mathrm{cos}\psi \right)}^{2}$ and k is the grain coordination number, here assumed to be equal to 8, for both unit cells. The distance between the spherical grain centres and flattened contacts is then given by ${R}_{\mathrm{sc}}=\sqrt{{R}_{\mathrm{s}}^{2}-{r}^{2}}$ (see Fig. 3d and h). We ignore clays not present within grain contacts, as these fall outside the load-bearing framework. Grain contact clay films are taken to consist of illite (see Sect. 4.1) and are disc or oval shaped, having the same top and bottom surface area as that of the contact ($\pi {r}^{2}$ for grain contacts B and $\pi {r}^{2}\sqrt{{\mathrm{sin}}^{2}\psi +{\chi }^{2}{\mathrm{cos}}^{2}\psi }$ for grain contacts A, cf. Fig. 3b and f) and a thickness z of 2.5 μm, following the clay film thicknesses estimated for Bleurswiller sandstone. Both unit cells incorporate eight inclined contacts (grain contacts A). In addition, the unit cell with $\psi =\frac{\pi }{6}$ incorporates two grain contacts parallel to the unit cell top and bottom (grain contacts B). We assume that a simplified sandstone sample consists of a mixture of the two unit cells (Fig. 3i and j). For present purposes, we assume 40% of the undeformed sandstone consists of the unit cell with $\psi =\frac{\pi }{6}$, and 60% consists of cells having distributed values of ψ, whereby the proportion of ψ-values linearly increases from 0 to 12% in the range $\psi =\frac{\pi }{6}$ to $\frac{\pi }{4}$. In the search for a suitable reference case, we considered a 50:50 ratio for cells with $\psi =\frac{\pi }{6}$ and $\psi =\frac{\pi }{6}$ to $\frac{\pi }{4}$, respectively. However, the 40:60 ratio showed closer agreement with the main body of data, so we chose that as our reference case. The choice and effect of the ψ distribution is discussed in Sect. 7.3. In line with the microstructural observations, we also assume that some of the unit cells miss intergranular clay films and the corresponding grain-to-grain contacts (i.e., inclined grain contacts A not intersecting with basal plane; cf. Fig. 3b) are open (i.e., no contact made) (see Sect. 4.1). For now, we assume that half of the unit cells miss four inclined clay films (Fig. 3b and f), and we will later discuss the effect of the choice of clay-free contact proportion.

Parameter values and assumptions for the reference case are listed in Table 2. The choice and effect of those parameter values will be discussed in Sect. 7.3.

### Assumed Deformation Processes

On the basis of the microstructural observations outlined in Sect. 4.1, we infer that inelastic deformation during triaxial compression of Bleurswiller sandstone is dominated by the following inelastic processes: rate-independent consolidation of intergranular clays; rate-dependent intergranular sliding; and rate-dependent intragranular cracking (stress corrosion cracking). The choice of the two unit cells implies that consolidation of intergranular clay films and intergranular sliding are either independent parallel processes (when $\frac{\pi }{6}<\psi <\frac{\pi }{4}$), or serial processes (when $\psi =\frac{\pi }{6}$), meaning that the slower process controls the rate of the other process. In describing intragranular cracking, we assume cracking only occurs vertically (Sect. 4.1), and hence only occurs from horizontal grain contacts B in the unit cell with $\psi =\frac{\pi }{6}$. This assumption is based on later calculations showing that the grain contact normal force, driving intragranular cracking, is largest on horizontal grain contact B, regardless of grain packing (i.e., ψ-values) and of the presence/absence of intergranular clay films. We differentiate between intragranular (meridional) splitting and intragranular multi-edge cracking, with reference to the dominant crack types seen in microstructures.

In the following, we first give general relations for the stresses acting on the unit cell and grain contact scales. We then develop a series of model components describing stress criteria and accompanying stress–strain and strain rate relations for the above processes assumed to dominate inelastic deformation during triaxial compression of our Bleurswiller sandstone. Finally, we integrate the different model components to evaluate the total inelastic deformation behaviour.

### Stress at the Unit Cell and Grain Contact Scales

To ensure homogeneous deformation in lateral directions, we choose the orientation of the maximum principal stress (${\sigma }_{1}$) in a way that it acts normal to the unit cell top and bottom. The intermediate (${\sigma }_{2}$) and minimum (${\sigma }_{3}$) principal stresses are assumed equal (${\sigma }_{2}={\sigma }_{3}$), to simulate axi-symmetric compression in a triaxial test or vertical loading of a horizontally bedded reservoir sandstone undergoing depletion with ${\sigma }_{2}\approx {\sigma }_{3}$ and act normal to the unit cell sides.

#### Stresses in the Unit Cell with ${\boldsymbol{\psi}}=\frac{{\boldsymbol{\pi}}}{6}$

Assuming that each unit cell supports the same stress (the externally applied stress) at its boundaries and ignoring the thickness of the intergranular clay films (i.e., taking $z\ll {R}_{\mathrm{a}}$ and ${\chi R}_{\mathrm{sc}}$, as observed; see Fig. 3c), the normal stress acting on the boundaries of the unit cell and those acting at grain contacts are related by:

4a $$\begin{array}{c}{\sigma }_{1}8{R}_{\mathrm{c}}^{2}{\mathrm{cos}}^{2}\psi =\pi {r}^{2}\left(2\eta \mathrm{cos}\alpha \sqrt{{\mathrm{sin}}^{2}\psi +{\chi }^{2}{\mathrm{cos}}^{2}\psi } {\widetilde{\sigma }}_{\mathrm{A}}+2\eta \mathrm{sin}\alpha \sqrt{{\mathrm{sin}}^{2}\psi +{\chi }^{2}{\mathrm{cos}}^{2}\psi } {\widetilde{\tau }}_{\mathrm{A}}+{2\widetilde{\sigma }}_{\mathrm{B}}\right), \end{array}$$

4b $$\begin{array}{c}{\sigma }_{3}8\sqrt{2}\chi {R}_{\mathrm{c}}^{2}\mathrm{sin}\psi \mathrm{cos}\psi =\pi {r}^{2}\sqrt{{\mathrm{sin}}^{2}\psi +{\chi }^{2}{\mathrm{cos}}^{2}\psi } \frac{1}{\sqrt{2}}\left(2\eta \mathrm{sin}\alpha {\widetilde{\sigma }}_{\mathrm{A}}-2\eta \mathrm{cos}\alpha {\widetilde{\tau }}_{\mathrm{A}}\right). \end{array}$$

Here, the subscripts to grain contact normal $\widetilde{\sigma }$ and shear $\widetilde{\tau }$ stresses denote the specific grain contact stress components (see Fig. 3c), and η takes a value of either 1 or 2, depending on whether the unit cell misses four inclined clay films in the grain contacts, or not (Fig. 3b). We assume that these force balance relations are satisfied within each cell-sized grain cluster during deformation. Ignoring cohesion in the clay films, normal and shear stresses acting on grain contact of type A are related as:

5 $$\begin{array}{c}{\widetilde{\tau }}_{\mathrm{A}}=\widetilde{\mu }{\widetilde{\sigma }}_{\mathrm{A},} \end{array}$$

where $\widetilde{\mu }$ is the friction coefficient at grain contacts. For Bleurswiller sandstone, $\widetilde{\mu }$ was estimated using the value of the macroscopic friction coefficient μ (∼0.61) obtained for simulated fault gouge material consisting mainly of quartz, with lesser amounts of feldspar and clay (i.e., Slochteren sandstone gouge), yielding $\widetilde{\mu }$-values equal to or less than 0.36 (Shinohara et al. 2024). On the other hand, wet shear tests were performed on layers of relatively pure (95 vol%) illite clay powder (simulated frictional wear material or “gouge” found in upper crustal faults) at room temperature, an effective normal stress of 40 MPa and a shear velocity of 1 μm/s point to a friction coefficient of illite powder from 0.22 to 0.30 at shear strains of 0.4–8.0 (Tembe et al. 2010). For present purposes, we choose an average friction coefficient (${\widetilde{\mu }}_{0}$), of 0.35 for clay-coated grain contacts at a reference sliding velocity of 1 μm/s, since our Bleurswiller sandstone consists of quartz, lesser amounts of feldspar and only some portions of the grain contacts are filled with clay films—implying that the $\widetilde{\mu }$-value is likely higher than the value for pure illite (0.22–0.30).

The grain contact normal stresses (${\widetilde{\sigma }}_{\mathrm{A}}$, ${\widetilde{\sigma }}_{\mathrm{B}}$) are now obtained by inserting Eq. 5 into Eqs. 4a and b, which gives:

6a $$\begin{array}{c}{\widetilde{\sigma }}_{\mathrm{A}}=\frac{8}{\pi \eta }{\left(\frac{{R}_{c}}{r}\right)}^{2}\frac{\chi }{\sqrt{{\mathrm{sin}}^{2}\psi +{\chi }^{2}{\mathrm{cos}}^{2}\psi }}\frac{\mathrm{sin}\psi \mathrm{cos}\psi }{\mathrm{sin}\alpha -\widetilde{\mu }\mathrm{cos}\alpha }{\sigma }_{3,} \end{array}$$

6b $$\begin{array}{c}{\widetilde{\sigma }}_{\mathrm{B}}=\frac{4}{\pi }{\left(\frac{{R}_{c}}{r}\right)}^{2}{\mathrm{cos}}^{2}\psi {\sigma }_{1}-\frac{8}{\pi }{\left(\frac{{R}_{c}}{r}\right)}^{2}\chi \left(\mathrm{cos}\alpha +\widetilde{\mu }\mathrm{sin}\alpha \right)\frac{\mathrm{sin}\psi \mathrm{cos}\psi }{\mathrm{sin}\alpha -\widetilde{\mu }\mathrm{cos}\alpha }{\sigma }_{3}. \end{array}$$

#### Stresses in the Unit Cell with $\frac{{\boldsymbol{\pi}}}{6}<{\boldsymbol{\psi}}<\frac{{\boldsymbol{\pi}}}{4}$

Again, assuming that each unit cell supports the same stress (the externally applied stress) at its boundaries, and ignoring the thickness of the intergranular clay films (i.e., taking $z\ll {R}_{\mathrm{a}}$ and ${R}_{\mathrm{b}}$, as observed in the microstructure; see Fig. 3g), the normal stress acting on the top/bottom planes and on the lateral planes of the unit cell, are related to the stresses acting at grain contacts by:

7a $$\begin{array}{*{20}c} {\sigma_{1}8R_{c}^{2} \cos^{2} \psi = \pi r^{2} \sqrt {\sin^{2}\psi+\chi^{2} \cos^{2}\psi}\left( {2\eta\cos\alpha\tilde{\sigma}_{{\mathrm{A}}} + 2\eta \sin \alpha\tilde{\tau}_{{\mathrm{A}}}}\right)}\\\end{array} ,$$

7b $$\begin{array}{c}{\sigma }_{3}8\sqrt{2}\chi {R}_{\mathrm{c}}^{2}\mathrm{sin}\psi \mathrm{cos}\psi =\pi {r}^{2}\sqrt{{\mathrm{sin}}^{2}\psi +{\chi}^{2}{\mathrm{cos}}^{2}\psi } \frac{1}{\sqrt{2}}\left(2\eta \mathrm{sin}\alpha {\widetilde{\sigma }}_{\mathrm{A}}-2\eta \mathrm{cos}\alpha {\widetilde{\tau }}_{\mathrm{A}}\right). \end{array}$$

Normal (${\widetilde{\sigma }}_{\mathrm{A}}$) and shear (${\widetilde{\tau }}_{\mathrm{A}}$) stresses are obtained by recasting Eqs. 7a and b, which gives:

8a $$\begin{array}{c}{\widetilde{\sigma }}_{\mathrm{A}}=\frac{4}{\pi \eta }{\left(\frac{{R}_{c}}{r}\right)}^{2}\frac{{\mathrm{cos}}^{2}\psi \mathrm{cos}\alpha }{\sqrt{{\mathrm{sin}}^{2}\psi +{\chi }^{2}{\mathrm{cos}}^{2}\psi }}{\sigma }_{1}+\frac{8}{\pi \eta }{\left(\frac{{R}_{c}}{r}\right)}^{2}\frac{\chi \mathrm{sin}\psi \mathrm{cos}\psi \mathrm{sin}\alpha }{\sqrt{{\mathrm{sin}}^{2}\psi +{\chi }^{2}{\mathrm{cos}}^{2}\psi }}{\sigma }_{3,} \end{array}$$

8b $$\begin{array}{c}{\widetilde{\tau }}_{\mathrm{A}}=\frac{4}{\pi \eta }{\left(\frac{{R}_{c}}{r}\right)}^{2}\frac{{\mathrm{cos}}^{2}\psi \mathrm{sin}\alpha }{\sqrt{{\mathrm{sin}}^{2}\psi +{\chi }^{2}{\mathrm{cos}}^{2}\psi }}{\sigma }_{1}-\frac{8}{\pi \eta }{\left(\frac{{R}_{c}}{r}\right)}^{2}\frac{\chi \mathrm{sin}\psi \mathrm{cos}\psi \mathrm{cos}\alpha }{\sqrt{{\mathrm{sin}}^{2}\psi +{\chi }^{2}{\mathrm{cos}}^{2}\psi }}{\sigma }_{3}. \end{array}$$

## Clay Consolidation and Intergranular Slip

### Consolidation Behaviour of Illite Within Grain Contacts

The drained, 1D consolidation (i.e., compaction) behaviour of illite powder has been investigated by performing long-term (months) oedometer tests on brine-saturated illite powder (purity 65%–85%) at room temperature, under effective axial stresses in the range of 5–180 MPa (Brown et al. 2017). Using their data, Pijnenburg and Spiers (2020) found that the inelastic normal strain rate $\dot{\widetilde{\varepsilon }}$ occurring within a grain contact illite film, due to 1D (film normal) consolidation accompanying an increase in grain contact normal stress at rate $\dot{\widetilde{\sigma }}$, is described by:

9 $$\begin{array}{c}\dot{\widetilde{\varepsilon }}=\frac{B}{\widetilde{\sigma }}\dot{\widetilde{\sigma }}, \end{array}$$

where B is a constant, estimated to be ~ 0.05 by Pijnenburg and Spiers (2020) (Table 2). The experimental conditions for the consolidation tests indicate that Eq. 9 is valid under the assumption that inelastic compaction of illite films in quartz/clastic grain contacts can be considered as 1D, instantly drained consolidation behaviour. This assumption seems reasonable since fluid drainage pathways are short (i.e., controlled by the small grain contact radius which is 10–100 μm in the Bleurswiller sandstone) and the frictional forces on the clay film surfaces imposed by the bounding quartz/clastic grain contacts limit radial expansion.

### Rate-Dependent Grain Boundary Sliding

On the basis of rate-and-state friction measurements made on clays and clay-bearing fault gouges, the grain boundary sliding friction coefficient $\widetilde{\mu }$ of clay-coated contacts of type A is expected to be rate-dependent and can be expressed in terms of grain boundary shearing velocity ${v}_{\mathrm{slip}}$ as:

10 $$\begin{array}{c}\widetilde{\mu }={\widetilde{\mu }}_{0}+{\widetilde{a}}_{\mu }\mathrm{ln}\left(\frac{{v}_{\mathrm{slip}}}{{v}_{\mathrm{slip}0}}\right), \end{array}$$

where ${\widetilde{\mu }}_{0}$ is the reference grain boundary friction coefficient measured at a reference velocity ${v}_{\mathrm{slip}0}$ and ${\widetilde{a}}_{\mu }$ is a rate-dependent coefficient constituting a direct effect in RSF terminology. Here, following Chen and Spiers (2016), we assume that the rate effect along grain boundary clay films is due to the transient effect of the RSF ${\widetilde{a}}_{\mu }$-value only, in contrast to more general rate sensitivity parameter (${\widetilde{a}}_{\mu }-{\widetilde{b}}_{\mu }$). This is justified for clay-bearing grain contacts, since the micromechanics of frictional sliding of gouge material suggests that another RSF parameter ${\widetilde{b}}_{\mu }$ is related to the evolution of porosity caused by dilation or compaction, which is likely negligible for clay films over a small shear displacement.

### Deviatoric Stress vs. Strain Behaviour due to Clay Consolidation and Grain Boundary Slip Operating in Serial and Parallel

In the unit cell with $\psi =\frac{\pi }{6}$, the model geometry requires that any consolidation of the clays present at contact type B must be accompanied by slip and consolidation of the clay films present at A (see Fig. 3b and c). This indicates that those processes are serially coupled, meaning that the slower process controls the rate of the other process. With reference to Fig. 3c, the kinematic constraints imposed by the unit cell geometry require that the inelastic normal strain rates at grain contacts ($\dot{\widetilde{\varepsilon }}$) developing due to consolidation of the clay films at A and B are related to the vertical inelastic strain rates (${\dot{\varepsilon }}_{1}^{\mathrm{inel}}$) and the horizontal inelastic strain rates (${\dot{\varepsilon }}_{\mathrm{2,3}}^{\mathrm{inel}}$) of the unit cell, through the following relationship:

11a $$\begin{array}{c}{\dot{\widetilde{\varepsilon }}}_{\mathrm{B}}=\frac{2{\chi R}_{\mathrm{c}}}{z}{\dot{\varepsilon }}_{1}^{\mathrm{inel}}, \end{array}$$

11b $$\begin{array}{c}{\dot{\widetilde{\varepsilon }}}_{\mathrm{A}}=\frac{2{R}_{\mathrm{c}}}{z}\left(\chi \mathrm{cos}\alpha {\dot{\varepsilon }}_{1}^{\mathrm{inel}}+\mathrm{sin}\alpha \mathrm{cos}\psi {\dot{\varepsilon }}_{\mathrm{2,3}}^{\mathrm{inel}}\right), \end{array}$$

11c $$\begin{array}{c}{v}_{\mathrm{slip}}=2{R}_{\mathrm{c}}\left(\chi \mathrm{sin}\alpha {\dot{\varepsilon }}_{1}^{\mathrm{inel}}-\mathrm{cos}\alpha \mathrm{cos}\psi {\dot{\varepsilon }}_{\mathrm{2,3}}^{\mathrm{inel}}\right). \end{array}$$

Combining Eqs. 10 and 11c, we obtain relations describing the intermediate ${\dot{\varepsilon }}_{2}^{\mathrm{inel}}$ and minimum ${\dot{\varepsilon }}_{3}^{\mathrm{inel}}$ principal inelastic strain rates (${\dot{\varepsilon }}_{2}^{\mathrm{inel}}={\dot{\varepsilon }}_{3}^{\mathrm{inel}}$), occurring in the unit cells in response to axial deformation at rate ${\dot{\varepsilon }}_{1}^{\mathrm{tot}}$. The result is given as:

12 $$\begin{array}{c}{\dot{\varepsilon }}_{\mathrm{2,3}}^{\mathrm{inel}}=\chi \frac{\mathrm{tan}\alpha }{\mathrm{cos}\psi }{\dot{\varepsilon }}_{1}^{\mathrm{inel}}-\frac{{v}_{0}}{2{R}_{\mathrm{c}}}\frac{1}{\mathrm{cos}\psi \mathrm{cos}\alpha }\mathrm{exp}\left(\frac{\widetilde{\mu }-{\widetilde{\mu }}_{0}}{{\widetilde{a}}_{\mu }}\right). \end{array}$$

At the same time, inserting Eq. 2 into Eq. 1, yields the following relations describing all three principal inelastic strain rates:

13a $$\begin{array}{c}{\dot{\varepsilon }}_{1}^{\mathrm{inel}}={\dot{\varepsilon }}_{1}^{\mathrm{tot}}-\frac{1}{E}{\dot{\sigma }}_{1}, \end{array}$$

13b $$\begin{array}{c}{\dot{\varepsilon }}_{\mathrm{2,3}}^{\mathrm{tot}}={\dot{\varepsilon }}_{\mathrm{2,3}}^{\mathrm{inel}}+\frac{\nu }{E}{\dot{\sigma }}_{1}. \end{array}$$

Recasting Eq. 13a, the axial stress rate ${\dot{\sigma }}_{1}$ is described as:

14 $$\begin{array}{c}{\dot{\sigma }}_{1}=\frac{{\dot{\varepsilon }}_{1}^{\mathrm{tot}}}{\frac{1}{E}+\frac{d{\varepsilon }_{1}^{\mathrm{inel}}}{d{\sigma }_{1}}}. \end{array}$$

Applying the chain rule $\frac{d{\varepsilon }_{1}^{\mathrm{inel}}}{d{\sigma }_{1}}=\frac{d{\varepsilon }_{1}^{\mathrm{inel}}}{d{\widetilde{\varepsilon }}_{\mathrm{B}}}\frac{d{\widetilde{\varepsilon }}_{\mathrm{B}}}{d{\widetilde{\sigma }}_{\mathrm{B}}}\frac{d{\widetilde{\sigma }}_{\mathrm{B}}}{d{\sigma }_{1}}$, along with Eqs. 11a, 9 and 6b which give $\frac{d{\varepsilon }_{1}^{\mathrm{inel}}}{d{\widetilde{\varepsilon }}_{\mathrm{B}}}=\frac{z}{2\chi {R}_{\mathrm{c}}}$, $\frac{d{\widetilde{\varepsilon }}_{\mathrm{B}}}{d{\widetilde{\sigma }}_{\mathrm{B}}}=\frac{B}{{\widetilde{\sigma }}_{\mathrm{B}}}$ and $\frac{d{\widetilde{\sigma }}_{\mathrm{B}}}{d{\sigma }_{1}}\approx \frac{4}{\pi }{\left(\frac{{R}_{\mathrm{c}}}{r}\right)}^{2}{\mathrm{cos}}^{2}\psi$ under triaxial conditions (i.e., constant ${P}_{\mathrm{c}}$), and noting that changes in ψ are negligible (i.e., ψ remains nearly constant) during deformation of the thin clay films at grain boundaries (i.e., z≪R; cf. Fig. 2c and d), now yields:

15 $$\begin{array}{c}\frac{d{\varepsilon }_{1}^{\mathrm{inel}}}{d{\sigma }_{1}}=\frac{zB}{\chi {\widetilde{\sigma }}_{\mathrm{B}}}\frac{2}{\pi }\frac{{R}_{\mathrm{c}}}{{r}^{2}}{\mathrm{cos}}^{2}\psi . \end{array}$$

Inserting Eq. 15 into Eq. 14 subsequently yields ${\dot{\sigma }}_{1}$. In turn, using Eqs. 13a and 14, ${\dot{\varepsilon }}_{1}^{\mathrm{inel}}$ is obtained. Hence, the total ${\dot{\varepsilon }}_{i}^{\mathrm{tot}}$, elastic ${\dot{\varepsilon }}_{i}^{\mathrm{el}}$ and inelastic ${\dot{\varepsilon }}_{i}^{\mathrm{inel}}$ strain rates, and the axial stress rate ${\dot{\sigma }}_{1}$ can all be obtained for the unit cell with $\psi =\frac{\pi }{6}$.

For the unit cell with $\frac{\pi }{6}<\psi <\frac{\pi }{4}$, the model geometry dictates that grain boundary slip and clay consolidation at contact A can occur in parallel (cf. Fig. 3g). Hence, the principal inelastic strain rates (${\dot{\varepsilon }}_{i}^{\mathrm{inel}}$), can be described as the sum of strain rates due to grain boundary slip ${\dot{\varepsilon }}_{i}^{\mathrm{slip}}$ and clay consolidation ${\dot{\varepsilon }}_{i}^{\mathrm{clay}}$:

16 $$\begin{array}{c}{\dot{\varepsilon }}_{i}^{\mathrm{inel}}={\dot{\varepsilon }}_{i}^{\mathrm{slip}}+{\dot{\varepsilon }}_{i}^{\mathrm{clay}}. \end{array}$$

With reference to Fig. 3g, strain rates due to grain boundary slip ${\dot{\varepsilon }}_{i}^{\mathrm{slip}}$ are now given as:

17a $$\begin{array}{c}{\dot{\varepsilon }}_{1}^{\mathrm{slip}}=\frac{{v}_{\mathrm{slip}}}{2\chi {R}_{\mathrm{c}}}\frac{\mathrm{sin}\alpha }{\mathrm{sin}\psi }, \end{array}$$

17b $$\begin{array}{c}{\dot{\varepsilon }}_{\mathrm{2,3}}^{\mathrm{slip}}=-\frac{{v}_{\mathrm{slip}}}{2{R}_{\mathrm{c}}}\frac{\mathrm{cos}\alpha }{\mathrm{cos}\psi }. \end{array}$$

The deformation due to grain boundary slip causes a change in ψ. The rate of change in ψ, as a result of grain boundary slip with a velocity ${v}_{\mathrm{slip}}$, is given by:

18 $$\begin{array}{c}\frac{d\psi }{dt}=-\frac{{v}_{\mathrm{slip}}}{{R}_{c}}\sqrt{{\mathrm{cos}}^{2}\alpha +{\left(\mathrm{sin}\alpha /\chi \right)}^{2}}. \end{array}$$

At the same time, inserting Eqs. 2 and 16 into Eq. 1 gives the relations describing the principal inelastic strain rates due to clay consolidation ${\dot{\varepsilon }}_{1}^{\mathrm{clay}}$ and the intermediate ${\dot{\varepsilon }}_{2}^{\mathrm{tot}}$ and minimum ${\dot{\varepsilon }}_{3}^{\mathrm{tot}}$ total strain rates:

19a $$\begin{array}{c}{\dot{\varepsilon }}_{1}^{\mathrm{clay}}={\dot{\varepsilon }}_{1}^{\mathrm{tot}}-{\dot{\varepsilon }}_{1}^{\mathrm{slip}}-\frac{1}{E}{\dot{\sigma }}_{1}, \end{array}$$

19b $$\begin{array}{c}{\dot{\varepsilon }}_{\mathrm{2,3}}^{\mathrm{tot}}={\dot{\varepsilon }}_{\mathrm{2,3}}^{\mathrm{slip}}+{\dot{\varepsilon }}_{\mathrm{2,3}}^{\mathrm{clay}}+\frac{\nu }{E}{\dot{\sigma }}_{1}. \end{array}$$

By recasting Eq. 19a, the axial stress rate ${\dot{\sigma }}_{1}$ is now described as:

20 $$\begin{array}{c}{\dot{\sigma }}_{1}=\frac{{\dot{\varepsilon }}_{1}^{\mathrm{tot}}-{\dot{\varepsilon }}_{1}^{\mathrm{slip}}}{\frac{1}{E}+\frac{d{\varepsilon }_{1}^{\mathrm{clay}}}{d{\sigma }_{1}}}. \end{array}$$

Using $\frac{d{\varepsilon }_{i}^{\mathrm{clay}}}{d{\sigma }_{1}}=\frac{d{\varepsilon }_{i}^{\mathrm{clay}}}{d{\widetilde{\varepsilon }}_{\mathrm{A}}}\frac{d{\widetilde{\varepsilon }}_{\mathrm{A}}}{d{\widetilde{\sigma }}_{\mathrm{A}}}\frac{d{\widetilde{\sigma }}_{\mathrm{A}}}{d{\sigma }_{1}}$, where, with reference to Fig. 3g and using Eqs. 9 and 8a, $\frac{d{\varepsilon }_{1}^{\mathrm{clay}}}{d{\widetilde{\varepsilon }}_{\mathrm{A}}}=\frac{z}{2\chi {R}_{\mathrm{c}}}\frac{\mathrm{cos}\alpha }{\mathrm{sin}\psi }$, $\frac{d{\varepsilon }_{\mathrm{2,3}}^{\mathrm{clay}}}{d{\widetilde{\varepsilon }}_{\mathrm{A}}}=\frac{z}{2{R}_{\mathrm{c}}}\frac{\mathrm{sin}\alpha }{\mathrm{cos}\psi }$, $\frac{d{\widetilde{\varepsilon }}_{\mathrm{A}}}{d{\widetilde{\sigma }}_{\mathrm{A}}}=\frac{B}{{\widetilde{\sigma }}_{\mathrm{A}}}$ and $\frac{d{\widetilde{\sigma }}_{\mathrm{A}}}{d{\sigma }_{1}}\approx \frac{4}{\pi \eta }{\left(\frac{{R}_{c}}{r}\right)}^{2}\frac{{\mathrm{cos}}^{2}\psi \mathrm{cos}\alpha }{\sqrt{{\mathrm{sin}}^{2}\psi +{\chi }^{2}{\mathrm{cos}}^{2}\psi }}$ under triaxial conditions (i.e., constant ${P}_{\mathrm{c}}$), and assuming the rate of change in ψ with respect to ${\sigma }_{1}$ is negligible (i.e., $\frac{\partial \psi }{\partial {\sigma }_{1}}\approx 0$), now yields:

21a $$\begin{array}{c}\frac{d{\varepsilon }_{1}^{\mathrm{clay}}}{d{\sigma }_{1}}=\frac{zB}{\chi {\widetilde{\sigma }}_{\mathrm{A}}}\frac{1}{\pi }\frac{{R}_{\mathrm{c}}}{{r}^{2}}\frac{{\mathrm{cos}}^{2}\psi {\mathrm{cos}}^{2}\alpha }{\mathrm{sin}\psi \sqrt{{\mathrm{sin}}^{2}\psi +{\chi }^{2}{\mathrm{cos}}^{2}\psi }}, \end{array}$$

21b $$\begin{array}{c}\frac{d{\varepsilon }_{\mathrm{2,3}}^{\mathrm{clay}}}{d{\sigma }_{1}}=\frac{zB}{{\widetilde{\sigma }}_{\mathrm{A}}}\frac{1}{\pi }\frac{{R}_{\mathrm{c}}}{{r}^{2}}\frac{\mathrm{cos}\psi \mathrm{sin}\alpha \mathrm{cos}\alpha }{\sqrt{{\mathrm{sin}}^{2}\psi +{\chi }^{2}{\mathrm{cos}}^{2}\psi }}. \end{array}$$

Inserting Eqs. 17a and 21a into Eq. 20 yields ${\dot{\sigma }}_{1}$. In turn, inserting Eq. 17a and the obtained value of ${\dot{\sigma }}_{1}$ into Eq. 19a, leads to an expression for ${\dot{\varepsilon }}_{1}^{\mathrm{clay}}$. At the same time, combining Eq. 21b and the value obtained for ${\dot{\sigma }}_{1}$ yields ${\dot{\varepsilon }}_{\mathrm{2,3}}^{\mathrm{clay}}=\frac{d{\varepsilon }_{\mathrm{2,3}}^{\mathrm{clay}}}{d{\sigma }_{1}}{\dot{\sigma }}_{1}$. Hence, with reference to Eqs. 17 to 21, expressions follow for the strain rate terms ${\dot{\varepsilon }}_{i}^{\mathrm{tot}}$, ${\dot{\varepsilon }}_{i}^{\mathrm{el}}$ and ${\dot{\varepsilon }}_{i}^{\mathrm{inel}}$ ($={\dot{\varepsilon }}_{i}^{\mathrm{slip}}+{\dot{\varepsilon }}_{i}^{\mathrm{clay}}$), and for the change in microstructure $\left(\frac{d\psi }{dt}\right)$ and axial stress rate ${\dot{\sigma }}_{1}$, for the unit cell with $\frac{\pi }{6}<\psi <\frac{\pi }{4}$.

## Time-Dependent Intragranular Splitting Near the Peak Stress

We now derive descriptions of the stress conditions and the strain increment associated with intragranular crack growth and grain failure, which, based on experimental observations in Shinohara et al. (2024), operates at differential stresses close to the peak stress. The analysis represents an extension of the rate-independent grain cracking model developed by Pijnenburg and Spiers (2020) such that it now accounts for time-dependent crack growth caused by stress corrosion mechanisms (Brantut et al. 2013, 2014; Heap et al. 2009a, b; Heap et al. 2009a, b; Heap et al. 2015; Pijnenburg et al. 2018; Shinohara et al. 2024). Assuming that strain increments associated with cracking are not dependent on the rate of crack growth, we employ the expressions of Pijnenburg and Spiers (2020) for the strain increment caused by grain cracking (see Sect. 6.3).

### Assumed Grain Contact Properties and Grain Failure Mode

**Fig. 4:**
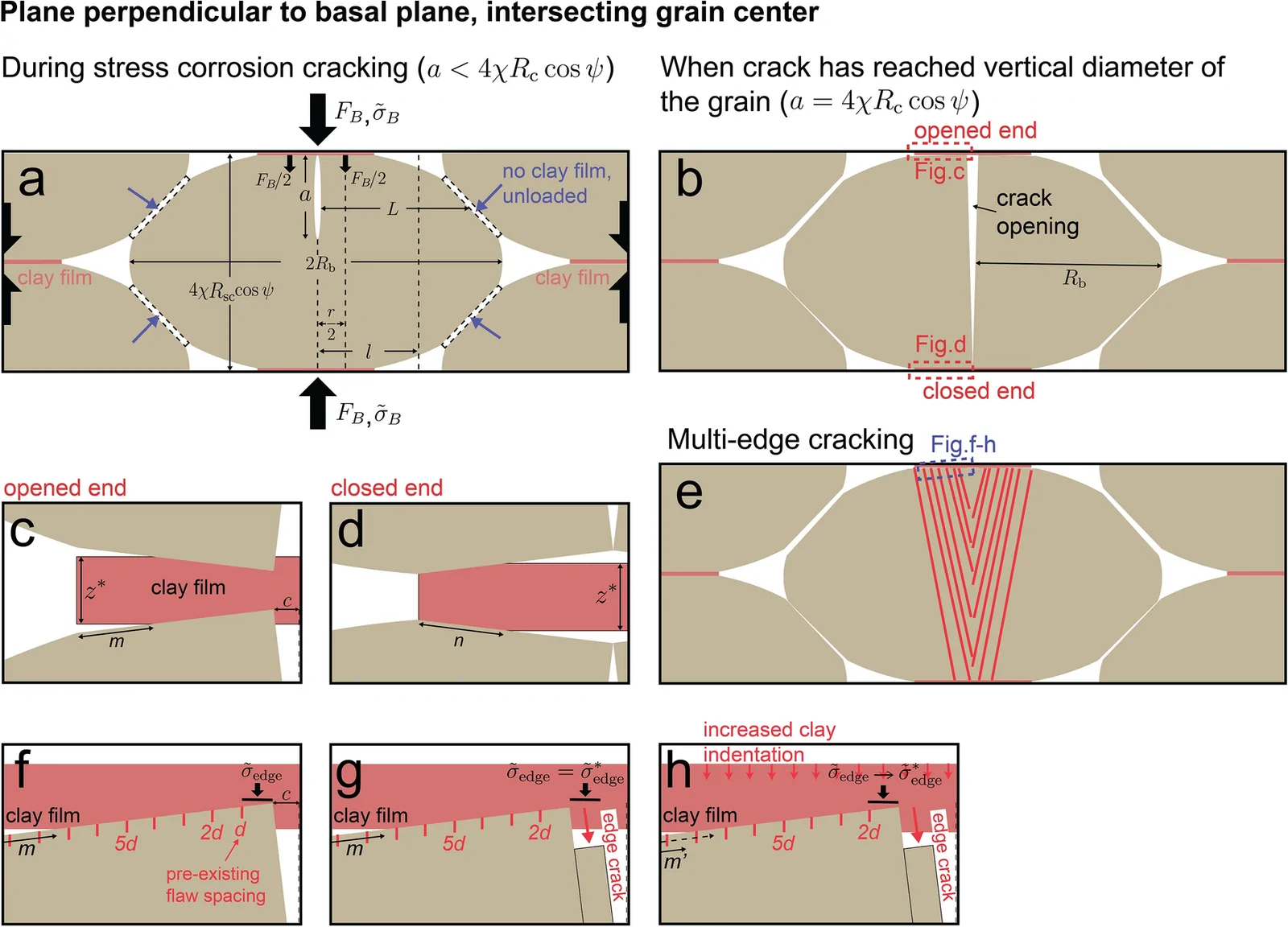
A view of a plane, perpendicular to a basal plane, illustrating the geometry and geometrical parameters considered for **a** meridional intragranular splitting caused by stress corrosion mechanisms, and associated strain increment by **b–d** a single meridional crack and **e–h** multi-edge cracks. The model is a modification of the time-independent meridional cracking model developed by Pijnenburg and Spiers (2020)

As a first approximation, we base this analysis on the following assumptions and simplifications:

1. Based on our microstructural observations that intragranular cracks dominantly emanate from grain-to-grain contacts (Fig. 1g–k), we assume such cracks are the cracking mechanism leading to strain. This is different from cracks that emanate from grain contact edges caused by tensile stress concentration at those locations (e.g., Shinohara et al. 2025a).
2. Within the flat grain contacts featured in our microstructural model, pre-existing stress concentrators and flaws are assumed present. These flaws are aligned in parallel to each other and orientated perpendicular to a basal plane (a flaw intersecting the grain centre with length a is schematically shown in Fig. 4). They have a characteristic mean spacing d of 5 μm, corresponding to microstructural observations of the typical spacing of such flaws (e.g., Fig. 1g-j).
3. The above grain contact roughness and flaw distribution are assumed to have negligible influence on the state of stress in the bulk of the grain, since the amplitude of grain contact asperities seen in the Bleurswiller sandstones is small (< 1% of the grain diameter). This means that the average grain contact stresses can be considered a sufficiently well-described state of contact stresses over the presently assumed, flat grain contact geometry.
4. The extension of grain contact flaws as cracks traversing the full grain diameter is assumed to be controlled by the normal stress ($\widetilde{\sigma }$) supported at grain contacts, in line with the particle splitting model of Kendall (1978) and grain crushing limit model by Sammis and Ben-Zion, (2008).
5. Since the highest normal load will be supported on grain contacts orientated perpendicular to ${\sigma }_{1}$, intragranular cracking will generally be easier at grain contact B in unit cell with $\psi =\frac{\pi }{6}$, compared to grain contact A in both unit cells with $\psi =\frac{\pi }{6}$ and $\frac{\pi }{6}<\psi <\frac{\pi }{4}$. In addition, since Kendall (1978) showed that confining pressure significantly increases the critical normal stress needed for propagation of grain contact flaws, intragranular cracking will be the easiest in unit cells with $\psi =\frac{\pi }{6}$ where inclined clay films are missing from some grain contacts, so that no traction is present (i.e., unit cells where effect of confining pressure on crack growth is negligible; Fig. 4a). Therefore, intragranular cracking is evaluated for this type of unit cell only.
6. Breaking grains are considered to split into two short struts by the growth of a central crack, with the growth of the crack being driven by the bending moment of the two struts, following the model for meridional cracking by Kendall (1978). With reference to Fig. 4a, the bending axis lies at distance $l=\lambda {R}_{\mathrm{b}}/2$ from the crack. The axial plane of bending has length $L=\kappa {R}_{\mathrm{b}}$.

### Assumed Grain Contact Properties and Grain Failure Mode

An expression for the strain rate associated with grain splitting is obtained by calculating the grain lifetime from the growth velocity $\dot{a}$ of the meridional crack. This is done by combining the conservation of energy for the deforming grain with the second law of thermodynamics to obtain the following grain-specific expression for thermal energy release rate or dissipation Δ (J/s),

22 $$\begin{array}{c}\Delta =\dot{W}-{\dot{f}}_{\mathrm{el}}-{\dot{f}}_{\mathrm{s}}=T{\dot{s}}_{i}\ge 0, \end{array}$$

where $\dot{W}$ is the rate of mechanical work supplied to the grain by the action of the axial contact force ${F}_{\mathrm{B}}$, ${\dot{f}}_{\mathrm{el}}$ is the rate at which elastic deformation energy is stored in the grain as the crack advances, ${\dot{f}}_{\mathrm{s}}$ is the rate at which energy is stored in the form of crack surface energy, T is absolute temperature and ${\dot{s}}_{i}$ is the rate of internal entropy production within the grain. With reference to the nomenclature defined in Fig. 4, this expression translates to:

23 $$\begin{array}{c}\Delta ={F}_{\mathrm{B}}\frac{d{x}_{\mathrm{v}}}{da}\dot{a}-\frac{d{f}_{\mathrm{el}}}{da}\dot{a}-\frac{d{f}_{\mathrm{s}}}{da}\dot{a}=T{\dot{s}}_{i}\ge 0, \end{array}$$

where ${x}_{\mathrm{v}}$ represents vertical (axial) compression of the grain by the action of the axial contact force ${F}_{\mathrm{B}}$. $\frac{d{f}_{\mathrm{el}}}{da}$ is decomposed into the rate at which elastic deformation energy is stored due to grain compression, per crack extension, $\frac{d{f}_{\mathrm{el}-\mathrm{c}}}{da}$, and the rate at which elastic deformation energy is stored due to bending, per crack extension, $\frac{d{f}_{\mathrm{el}-\mathrm{b}}}{da}$. However, the compression component of elastic deformation energy does not change with crack length; hence, $\frac{d{f}_{\mathrm{el}}}{da}= \frac{d{f}_{\mathrm{el}-\mathrm{c}}}{da}+\frac{d{f}_{\mathrm{el}-\mathrm{b}}}{da} \approx \frac{d{f}_{\mathrm{el}-\mathrm{b}}}{da}$ (Kendall 1978).

For a linear elastic material, ${F}_{\mathrm{B}}\frac{d{x}_{\mathrm{v}}}{da}=2\frac{d{f}_{\mathrm{el}}}{da}\approx 2\frac{d{f}_{\mathrm{el}-\mathrm{b}}}{da}$ (e.g., Kendall 1978). Hence, Eq. 23 now becomes:

24 $$\begin{array}{c}\Delta =\frac{d{f}_{\mathrm{el}-\mathrm{b}}}{da}\dot{a}-\frac{d{f}_{\mathrm{s}}}{da}\dot{a}=T{\dot{s}}_{i}\ge 0. \end{array}$$

Using the Bernoulli–Euler expression, the elastic energy due to bending is given as ${f}_{\mathrm{el}-\mathrm{b}}=a{M}^{2}/({E}_{\mathrm{g}}I)$, where, with reference to Fig. 4, $M=\frac{{F}_{\mathrm{B}}}{2}\left(l-\frac{r}{2}\right)$ is the bending moment about the crack, per strut, ${E}_{\mathrm{g}}$ is the Young’s modulus of the grain and $I=\frac{1}{12}b{L}^{3}$ is the corresponding moment of inertia about the axial plane of bending. We assume that the bending axis l lies at distance $l=\lambda {R}_{b}/2$, and the axial plane of bending has length $L=\kappa {R}_{b}$, where λ and κ are geometrical constants that are both equal to 1 when the grain geometry is rectangular (Kendall 1978). The ellipsoidal geometry of our model grains implies that the values for λ and κ are smaller than 1; hence, we employed $\lambda =\kappa =0.5$ for the following analysis. The effect of the choice of these values will be discussed in Sect. 7.3. The rate of release of mechanical energy per unit crack extension is then given by:

25 $$\begin{array}{c}\frac{d{f}_{\mathrm{el}-\mathrm{b}}}{da}=\frac{3}{4}{F}_{\mathrm{B}}^{2}\frac{{\left(\lambda {R}_{\mathrm{b}}-r\right)}^{2}}{{E}_{\mathrm{g}}b{\left(\kappa {R}_{\mathrm{b}}\right)}^{3}}. \end{array}$$

With the relation $\frac{d{f}_{\mathrm{s}}}{da}=b{R}_{\mathrm{env}}$ for the rate of the total free energy stored in the crack surface area per crack extension, Eq. 24 now yields:

26 $$\begin{array}{c}\Delta =\left[\frac{3}{4}{F}_{\mathrm{B}}^{2}\frac{{\left(\lambda {R}_{\mathrm{B}}-r\right)}^{2}}{{E}_{\mathrm{g}}b{\left(\kappa {R}_{\mathrm{B}}\right)}^{3}}-b{R}_{\mathrm{env}}\right]\dot{a}=T{\dot{s}}_{i}\ge 0, \end{array}$$

where the crack surface energy ${R}_{\mathrm{env}} (\mathrm{J}/{\mathrm{m}}^{2})$ is two times the specific interfacial energy ${\gamma }_{\mathrm{env}}$ of fluid-filled cracks. Written in terms of dissipation per unit length of crack front (b), as opposed to per grain, this now gives:

27 $$\begin{array}{c}\left[\frac{3}{4}{F}_{\mathrm{B}}^{2}\frac{{\left(\lambda {R}_{\mathrm{b}}-r\right)}^{2}}{{E}_{\mathrm{g}}{b}^{2}{\left(\kappa {R}_{\mathrm{b}}\right)}^{3}}-{R}_{\mathrm{env}}\right]\dot{a}=T{\dot{s}}_{i}\ge 0. \end{array}$$

Here, the square-bracketed term represents the difference between i) the mechanical energy release rate per unit crack extension (cf. Rice 1978), i.e., the crack extension force per unit crack front, given:

28 $$\begin{array}{c}G= \frac{3}{4}{F}_{\mathrm{B}}^{2}\frac{{\left(\lambda {R}_{\mathrm{b}}-r\right)}^{2}}{{E}_{\mathrm{g}}{b}^{2}{\left(\kappa {R}_{\mathrm{b}}\right)}^{3}}\end{array}$$

and ii) the resistence to crack extension offered by the force ${R}_{\mathrm{env}}$, which expresses the work done in increasing crack surface area per unit crack extension and per unit crack front. Eq. 27 can accordingly be written as the thermodynamic force × flux relation:

29 $$\begin{array}{c}\left(G-{R}_{\mathrm{env}}\right)\dot{a}=T{\dot{s}}_{i}\ge 0. \end{array}$$

This equation is identical to the general relation for crack growth obtained by Rice (1978), but G and ${R}_{\mathrm{env}}$ are now specified for meridional grain cracking.

For present purposes, we focus on the case of subcritical crack growth by stress corrosion. This has been extensively studied and characterized in glasses and quartz (Atkinson 1984; Darot and Guéguen 1986; Lawn 1993), and the crack growth velocity $\dot{a}=v$ is typically related to driving force $g= G-{R}_{\mathrm{env}}$ via relationships of the form:

30 $$\begin{array}{c}v={v}_{\mathrm{cr}0}\mathrm{exp}\left[\beta \left(G-{R}_{\mathrm{env}}\right)\right],\end{array}$$

where ${v}_{\mathrm{cr}0}$ and β are constants at constant temperature and chemical conditions.

We assume that the two grain halves open up and strain is incremented when the crack length reaches the vertical diameter of the grain ($4\chi {R}_{\mathrm{sc}}\mathrm{cos}\psi$), i.e., when the crack fully splits the grain into two halves (Fig. 4b).

### Strain due to Grain Splitting

We employed the equations for strain increment due to meridional cracking of grains proposed by Pijnenburg and Spiers (2020). In contrast to their model where grains are spherical, grains in our model are ellipsoidal, resulting in an additional term $\chi =({R}_{\mathrm{a}}/{R}_{\mathrm{b}})$ in our equations. Nonetheless, only a brief summary is included here, since the remaining equations are identical.

When the growing crack has extended across the full vertical diameter of the grain, the crack is assumed to open preferentially and permanently towards open pore space (i.e., into the space where clay films are missing; see blue, dashed rectangular space in Fig. 4a and geometry where crack is open in Fig. 4b). With reference to Fig. 4b–d, the vertical strain increment of the unit cell due to intragranular splitting is estimated via (Pijnenburg and Spiers 2020):

31 $$\begin{array}{c}d{\varepsilon }_{1}^{\mathrm{cr},\mathrm{s}}=\frac{c\left(n-m\right)}{4{\chi }^{2}{R}_{\mathrm{sc}}^{2}},\end{array}$$

where c is half crack opening upon the intragranular splitting and n and (1-m) are the contact length between grain and indented clay film at the open (Fig. 4c) and closed (Fig. 4d) ends of the crack, respectively. Since intragranular splitting is considered for laterally unconfined grains (cf. Fig. 4a and b), crack opening will not cause any lateral strains, so that $d{\varepsilon }_{3}^{\mathrm{cr},\mathrm{s}}=0$. The values of c, n and m are obtained by balancing the forces supported by the grain contacts at both ends of the crack prior to opening, versus those supported after opening. Those force balance equations are given as (Pijnenburg and Spiers 2020):

32a $$\begin{array}{c}1=\frac{4\chi B{R}_{\mathrm{c}}{z}^{*}}{c\pi {r}^{2}}\left[\frac{2\chi B{R}_{\mathrm{c}}{z}^{*}}{c}\left(1-\mathrm{exp}\left(\frac{c\left(r/2-m \right)}{\chi B{R}_{\mathrm{c}}{z}^{*}}\right)\right)+r\mathrm{exp}\left(\frac{c\left(r-m\right)}{\chi B{R}_{\mathrm{c}}{z}^{*}}\right)-2m\right]. \end{array}$$

32b $$\begin{array}{c}1=\frac{4\chi B{R}_{\mathrm{c}}{z}^{*}}{c\pi {r}^{2}}\left[\frac{2\chi B{R}_{\mathrm{c}}{z}^{*}}{c}\left(\mathrm{exp}\left(\frac{cn}{\chi B{R}_{\mathrm{c}}{z}^{*}}\right)-\mathrm{exp}\left(\frac{c\left(n-r/2\right)}{\chi B{R}_{\mathrm{c}}{z}^{*}}\right)\right)-r\right]. \end{array}$$

In addition, assuming that the energy required to form new crack surface is provided by elastic energy released, as described by Kendall’s criterion (Kendall 1978), the external work increment per unit volume ($d{W}_{\mathrm{ext}}$) done through deforming the unit cell by intragranular splitting must be approximately equal to the internal work increment per unit volume ($d{W}_{\mathrm{int}}$) done (dissipated) by indenting all clay films at contacts B within the cell. For the presently considered deviatoric case with zero $d{\varepsilon }_{3}^{\mathrm{cr},\mathrm{s}}$, $d{W}_{\mathrm{ext}}$ is given by (Pijnenburg and Spiers 2020):

33 $$\begin{array}{c}d{W}_{\mathrm{ext}}=d{\varepsilon }_{1}^{\mathrm{cr},\mathrm{s}}{\sigma }_{1}^{*}, \end{array}$$

where ${\sigma }_{1}^{*}$ is the axial stress when crack length has reached the vertical diameter of the grain. $d{W}_{\mathrm{int}}$, which is the sum of the work done to achieve indentation of a clay film at the open and closed ends of the crack, is given as (Pijnenburg and Spiers 2020):

34 $$d{W}_{\mathrm{int}}=\frac{{\left({z}^{*}B\right)}^{2}{\widetilde{\sigma }}_{B}^{*}}{c\sqrt{3}{R}_{\mathrm{c}}^{2}}\left[\mathrm{exp}\left(\frac{c(r/2-m)}{\chi B{R}_{\mathrm{c}}{z}^{*}}\right)\left(\frac{2m-r}{2}+\frac{2\chi B{R}_{\mathrm{c}}{z}^{*}}{c}\right)+\mathrm{exp}\left(\frac{c(r-m)}{\chi B{R}_{\mathrm{c}}{z}^{*}}\right)\left(\frac{c({r}^{2}-rm)}{2\chi B{R}_{\mathrm{c}}{z}^{*}}-\frac{r}{2}\right)+m-\frac{2\chi B{R}_{\mathrm{c}}{z}^{*}}{c}+\mathrm{exp}\left(\frac{c\left(n-r/2\right)}{\chi B{R}_{\mathrm{c}}{z}^{*}}\right)\left(\frac{2\chi B{R}_{\mathrm{c}}{z}^{*}}{c}-n+\frac{r}{2}\right)+\frac{r}{2}+\mathrm{exp}\left(\frac{cn}{\chi B{R}_{\mathrm{c}}{z}^{*}}\right)\left(n-\frac{2\chi B{R}_{c}{z}^{*}}{c}\right)\right]. ($$

**Fig. 5:**
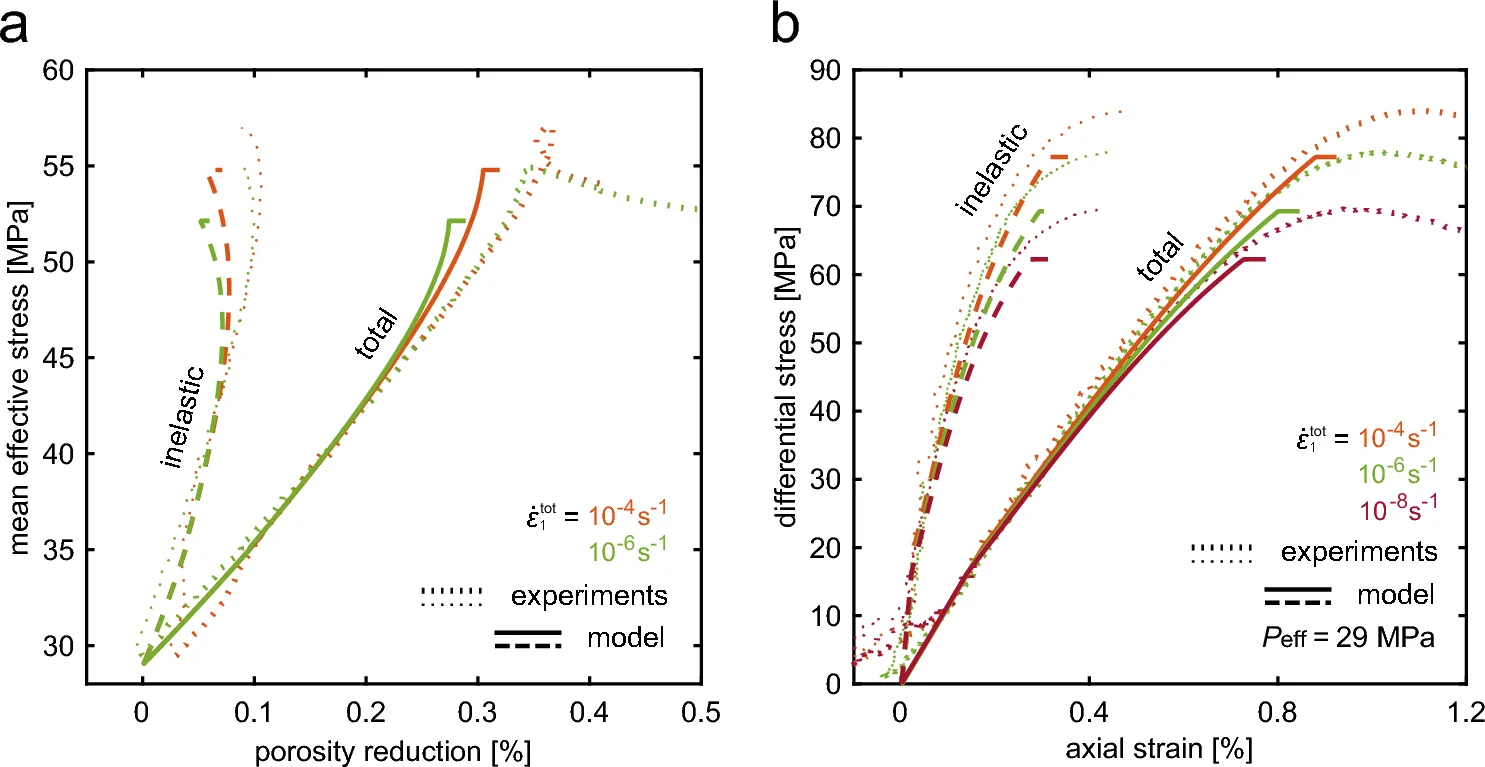
Plots showing the modelled **a** mean effective stress P versus inelastic $\Delta {\phi }_{\mathrm{inel}}$ and total $\Delta {\phi }_{\mathrm{tot}}$ porosity reduction and **b** differential stress Q versus total ${\varepsilon }_{1}^{\mathrm{tot}}$ and inelastic ${\varepsilon }_{1}^{\mathrm{inel}}$ axial strains, compared with corresponding mechanical data obtained in laboratory experiments on Bleurswiller sandstone samples used in Shinohara et al. (2024). The modelled and experimental data show roughly similar behaviour. Note that strain rate was varied per model run, as indicated. Model results are plotted for the reference case assumptions and parameter choices identified in the text

By employing Eqs. 32–34, the values of m, n, $d{W}_{\mathrm{ext}}$ and $d{W}_{\mathrm{int}}$, corresponding to a range of c-values between 10 nm and 5 μm, are numerically obtained. The values obtained for the half opening c, at which the external and internal work increments per unit volume are balanced ($d{W}_{\mathrm{ext}}=d{W}_{\mathrm{int}}$), yield 0.13–0.14 μm for imposed total axial strain rates between ${10}^{-4}$ and ${10}^{-8} \, {\mathrm{s}}^{-1}$ (the corresponding mechanical data is presented in Fig. 5). This range of c corresponds to full crack opening values (2c) in the range 0.26–0.28 μm. These values are comparable, although perhaps on the low side, to the crack openings typically observed in micrographs of our deformed samples (0.1–5 μm; Fig. 1g–k). The corresponding vertical strain increments due to splitting, $d{\varepsilon }_{1}^{\mathrm{cr},\mathrm{s}}$, ranged from 0.01 to 0.02%. Note that, in deriving Eqs. 32a and b, it was assumed that the values of m and n lie in the ranges 0<m<r/2 and r/2<n<r (Pijnenburg and Spiers 2020). We confirmed that this is indeed the case, for all the model runs conducted in this study.

When a meridional crack, with a half opening width of c, is formed, the normal stress acting around the crack is enhanced where the clay film is indented the most, particularly around the open crack end (Fig. 4f). Here, the normal stress ${\widetilde{\sigma }}_{\mathrm{edge}}$ will tend to extend the first neighbouring contact flaw located at a distance d (5 μm) from the open crack end (cf. Fig. 4f), causing an edge crack to run parallel to the initial crack surface. Pijnenburg and Spiers (2020) demonstrated that the enhanced normal stress ${\widetilde{\sigma }}_{\mathrm{edge}}$ acting on the edge adjacent to the initial meridional crack is higher than normal stress needed for the edge cracking to occur ${\widetilde{\sigma }}_{\mathrm{edge}}^{*}$, when contact flaws have a spacing d of 3 μm (Thouless et al. 1987). Since ${\widetilde{\sigma }}_{\mathrm{edge}}^{*}$ decreases with increasing flaw spacing (Thouless et al. 1987), ${\widetilde{\sigma }}_{\mathrm{edge}}>{\widetilde{\sigma }}_{\mathrm{edge}}^{*}$ for our Bleurswiller sandstone (d=5 μm), meaning that initial meridional splitting and crack opening is immediately followed by edge cracking (cf. Fig. 4g). We assume that this is also the case for our rate-dependent meridional cracking model.

When edge cracking has occurred, the two grain slices with width d, situated on both sides of the initial crack, will break off and move into the spaces opened by the initial, intragranular split (cf. Fig. 4g). The broken-off slices will not support load unless they fill the gap sufficiently. The load will now be supported on the remaining contact area, with the largest normal stress, found at the now most-uplifted edge (cf. Fig. 4h), again exceeding normal stress required for the edge cracking to occur (Pijnenburg and Spiers 2020). This results in edge cracking propagation from the next pair of flaws. Hence, at constant stress, multiple stages of edge cracking are expected. This process will continue until the space opened by the initial split is filled, such that the broken-off slices will start to support load. During deviatoric loading, the axial strain increment due to this multi-edge cracking is given by (Pijnenburg and Spiers 2020):

35 $$\begin{array}{c}d{\varepsilon }_{1}^{\mathrm{cr},\text{ m}}\approx \frac{\left(1-{\phi }_{\mathrm{cr}}\right)\left(c\chi {R}_{\mathrm{c}}+\frac{c}{{R}_{\mathrm{c}}}{\left(r-n\right)}^{2}\right)}{2r{\chi R}_{\mathrm{c}}}, \end{array}$$

while $d{\varepsilon }_{3}^{\mathrm{cr},\mathrm{m}}$ is zero, since this multi-edge cracking is considered for laterally unconfined grains hence opening will not induce any lateral strains.

Following this model, the total inelastic strain increment $d{\varepsilon }_{1}^{cr},$ developing at the vertical stress corresponding to initial intragranular splitting, is the sum of the strain increment caused by initial splitting $d{\varepsilon }_{1}^{\mathrm{cr},\mathrm{s}}$ and those resulting from subsequent multi-edge cracking $d{\varepsilon }_{1}^{\mathrm{cr},\mathrm{m}}$ occurring at constant stress; hence, $d{\varepsilon }_{1}^{\mathrm{cr}}=d{\varepsilon }_{1}^{\mathrm{cr},\mathrm{s}}+d{\varepsilon }_{1}^{\mathrm{cr},\mathrm{m}}$.

## Multi-mechanism Model Integration

We have developed a series of models for inelastic deformation in Bleurswiller sandstone, based on the grain-scale processes observed, inferred and assumed to operate during deviatoric loading of Bleurswiller sandstone at ${P}_{\mathrm{eff}}=29 \, \mathrm{MPa}$ and $T=100 \, ^{\circ}{\rm C}$ in the experiments in Shinohara et al. (2024). We have obtained equations to describe inelastic stress–strain behaviour caused by processes such as (1) rate-independent intergranular clay consolidation, (2) rate-dependent grain boundary slip and (3) intragranular stress corrosion cracking (i.e., meridional splitting, followed by multi-edge cracking at assumed constant stress). Clay consolidation and grain boundary slip occur either in parallel in the unit cell with $\frac{\pi }{6}<\psi <\frac{\pi }{4}$, or in series (i.e., slower process controls the rate of the other process) in the unit cell with $\psi =\frac{\pi }{6}$.

The contribution of each of the above processes to the integrated inelastic deformation behaviour of the model sandstone was assumed to be determined by the distribution of ψ. For present purposes, we chose the reference case distribution (Sect. 4.2) where 40% of the undeformed sandstone consists of the unit cell with ${\psi }_{0}=\frac{\pi }{6}$, where ${\psi }_{0}$ is the equivalent grain contact angle ψ at the start of deformation, and 60% with values of ${\psi }_{0}$ distributed so that frequency of ${\psi }_{0}$-values, in intervals of 1 degree (= π/180 radians), linearly increases from zero to 14% from ${\psi }_{0}=\frac{\pi }{6}$ to $\frac{\pi }{4}$. Following this distribution, a set of discrete values of ${\psi }_{0}$ from ${30}^{\circ }$ to ${44}^{\circ }$, with a spacing of ${1}^{\circ }$ (i.e., $\psi ={30}^{\circ }$,${31}^{\circ }$,${32}^{\circ }$, …,${44}^{\circ }$), was employed. Recall that elastic deformation is considered to develop independently (i.e., additively, cf. Eq. 1) and is described through the linear elasticity relations given in Eqs. 2a and 2b.

In the unit cell with ${\psi }_{0}=\frac{\pi }{6}$, Eq. 6b implies that grain contacts B can only exist (i.e., ${\widetilde{\sigma }}_{\mathrm{B}}>0$) when ${\sigma }_{1}$ is sufficiently larger than ${\sigma }_{3}$. To numerically find ${\sigma }_{1}$ ($>{{\sigma }_{2}=\sigma }_{3}$) at the start of axial straining (i.e., initial condition), we first calculated the effect of hydrostatic loading (i.e., ${\sigma }_{1}={\sigma }_{2}={\sigma }_{3}$) on this unit cell, assuming that shear stress on grain contacts A is zero (Pijnenburg and Spiers 2020) up to a typical lithostatic (assumed hydrostatic) stress for a depleted reservoir of 29 MPa (in this case, the Groningen gas reservoir). Then, we imposed a prescribed axial strain rate, while setting ${\dot{\varepsilon }}_{i}^{\mathrm{el}}$ to zero, to obtain an initial value of ${\sigma }_{1}$. This resulted in a differential stress level ($Q={\sigma }_{1}-{\sigma }_{3}$) of 21–23 MPa at the start of axial straining in the unit cell with ${\psi }_{0}=\frac{\pi }{6}$, at axial strain rates between ${10}^{-4}$ and ${10}^{-8} \, {\mathrm{s}}^{-1}$. At lower Q levels, we assume that the stress–strain behaviour of the unit cell is purely elastic.

We now integrate the above models by compiling the full set of equations using MATLAB software to calculate the implied, integrated stress versus elastic and inelastic strain behaviour. We assume that axial strain and confining pressure are uniformly distributed in the integrated, simplified sandstone aggregate (cf. Fig. 3i), meaning that an axial strain and a confining pressure, imposed on the aggregate, are applied to each unit cell. This implies that resultant axial stress and lateral strains are distributed from cell to cell, depending on values of ${\psi }_{0}$. The axial stress and lateral strains at the aggregate scale are hence averaged values.

One assumption made in our analysis is that the strain increment caused by stress corrosion cracking occurs instantaneously upon arrival of a meridional crack at the opposite side of a failing grain. Since the experiments on Bleurswiller sandstone (Shinohara et al. 2024) suggest that stress corrosion cracking controls inelastic deformation close to and at the peak stress, the integrated behaviour of the present model is explored until the instantaneous strain increment caused by stress corrosion cracking occurs. This means that the integrated behaviour obtained below the peak stress in a simulated triaxial test, for example, is caused solely by elastic deformation plus inelastic deformation caused by clay consolidation and intergranular slip.

### Model Comparison with Experimental Data

The modelled mechanical behaviour at ${P}_{\mathrm{c}}=29 \, \mathrm{MPa}$ and at axial strain rates on the order of ${10}^{-4}$ to ${10}^{-8} \, {\mathrm{s}}^{-1}$ is shown in Fig. 5a and b, for the reference case assumptions and parameter values (Table 2). We present modelled mean effective stress ($P= \left[{\sigma }_{1}+{\sigma }_{2}+{\sigma }_{3}\right]/3=\left[{\sigma }_{1}+2{\sigma }_{3}\right]/3$) versus inelastic ($\Delta {\phi }_{\mathrm{inel}}\approx {\varepsilon }_{1}^{\mathrm{inel}}+2{\varepsilon }_{3}^{\mathrm{inel}}$) and total ($\Delta {\phi }_{\mathrm{tot}}\approx {\varepsilon }_{1}^{\mathrm{el}}+2{\varepsilon }_{3}^{\mathrm{el}}+\Delta {\phi }_{\mathrm{inel}}$) porosity reduction curves (Fig. 5a), as well as differential stress Q versus total ${\varepsilon }_{1}^{\mathrm{tot}}$ and inelastic strain ${\varepsilon }_{1}^{\mathrm{inel}}$ curves (Fig. 5b). Direct comparisons are made in Fig. 5 with the corresponding experimental data obtained for Bleurswiller sandstone.

The modelled and experimentally obtained P versus $\Delta {\phi }_{\mathrm{inel}}$ and $\Delta {\phi }_{\mathrm{tot}}$ curves, and Q versus ${\varepsilon }_{1}^{\mathrm{tot}}$ and ${\varepsilon }_{1}^{\mathrm{inel}}$ curves, are typically similar within 0.05% porosity reduction or axial strain, up to the modelled peak stress at a given strain rate—ignoring the scattering in the experimental data at $P<36 \, \mathrm{MPa}$ or $Q<20 \, \mathrm{MPa}$ (Fig. 5a and b). The model shows quasi-linear stress vs. porosity reduction behaviour up to P=43–$47\, \mathrm{MPa}$, followed by an upward turn (i.e., increase in slope of the curves; Fig. 5a), which occurred at lower differential stress with decreasing axial strain rate, in line with the experimental data. This upward turn is followed by an instantaneous strain increment (i.e., compaction) at a constant P, characterizing the peak stress. In all modelled simulations, the peak stress was reached at 0.72%–0.88% axial strain (Fig. 5b). The modelled peak differential stress (i.e., the terminal stress at which cracking occurs) decreased with decreasing axial strain rate, in line with experimental observations. The lowering in the modelled peak stress that accompanies the reduction in axial strain rates from ${10}^{-4}$ to ${10}^{-8} \, {\mathrm{s}}^{-1}$ ($3.8 \, \mathrm{MPa}$ per decrease in strain rate by one order of magnitude) is comparable to that observed in the experimentals ($3.7 \, \mathrm{MPa}$ per decrease in strain rate by one order of magnitude).

On the other hand, the modelled peak differential stress is generally lower than that observed in the corresponding experiments by 7–9 MPa (Fig. 5b). This underestimation may be due to (1) the value chosen for the specific interfacial energy ${\gamma }_{\mathrm{env}}$ of fluid-filled cracks within quartz/clastic grains ($1.46 \, \mathrm{J}/{\mathrm{m}}^{2}$) and/or (2) the multi-edge cracking that instantaneously follows meridional cracking. The ${\gamma }_{\mathrm{env}}$-value chosen is based on a molecular dynamics simulation for quartz (Du and De Leeuw 2006) and falls within a poorly constrained range of experimentally obtained values (~ 0.4–11.5 $\mathrm{J}/{\mathrm{m}}^{2}$) (Atkinson and Meredith 1987). The choice of ${\gamma }_{\mathrm{env}}$-value will be discussed in Sect. 7.3. In addition, the strain increment caused by stress corrosion cracking seems to be underestimated, compared to experimental data, despite our reference case assumption that cracking can occur in 50% of the unit cells with ${\psi }_{0}=\frac{\pi }{6}$ (i.e., the unit cells that miss intergranular clay films; Fig. 4). This underestimation persists even if we assume cracking can occur in all (100%) of the unit cells with ${\psi }_{0}=\frac{\pi }{6}$, in which case the associated strain increment will be doubled compared to our reference case. Such underestimation may be caused by the fact that we ignore any stress corrosion cracking and associated strain increment at inclined grain contacts (i.e., grain contact A) in the unit cells with $\frac{\pi }{6}<{\psi }_{0}<\frac{\pi }{4}$.

Despite these various, modest discrepancies, the modelled and experimental data do show general agreement, taking the reference case and using parameter values listed in Table 2. Sensitivity analyses of computed behaviour versus the parameters used and assumptions made (e.g., proportion of unit cells with ${\psi }_{0}=\frac{\pi }{6}$) (Sect. 7.3) further shows that the behaviour modelled using reference case parameter values yields one of the best fits to the experimental data. This implies that the mechanical behaviour of Bleurswiller sandstone can be largely accounted for by the assumed mechanisms.

### Behaviour of Different Unit Cells

**Fig. 6:**
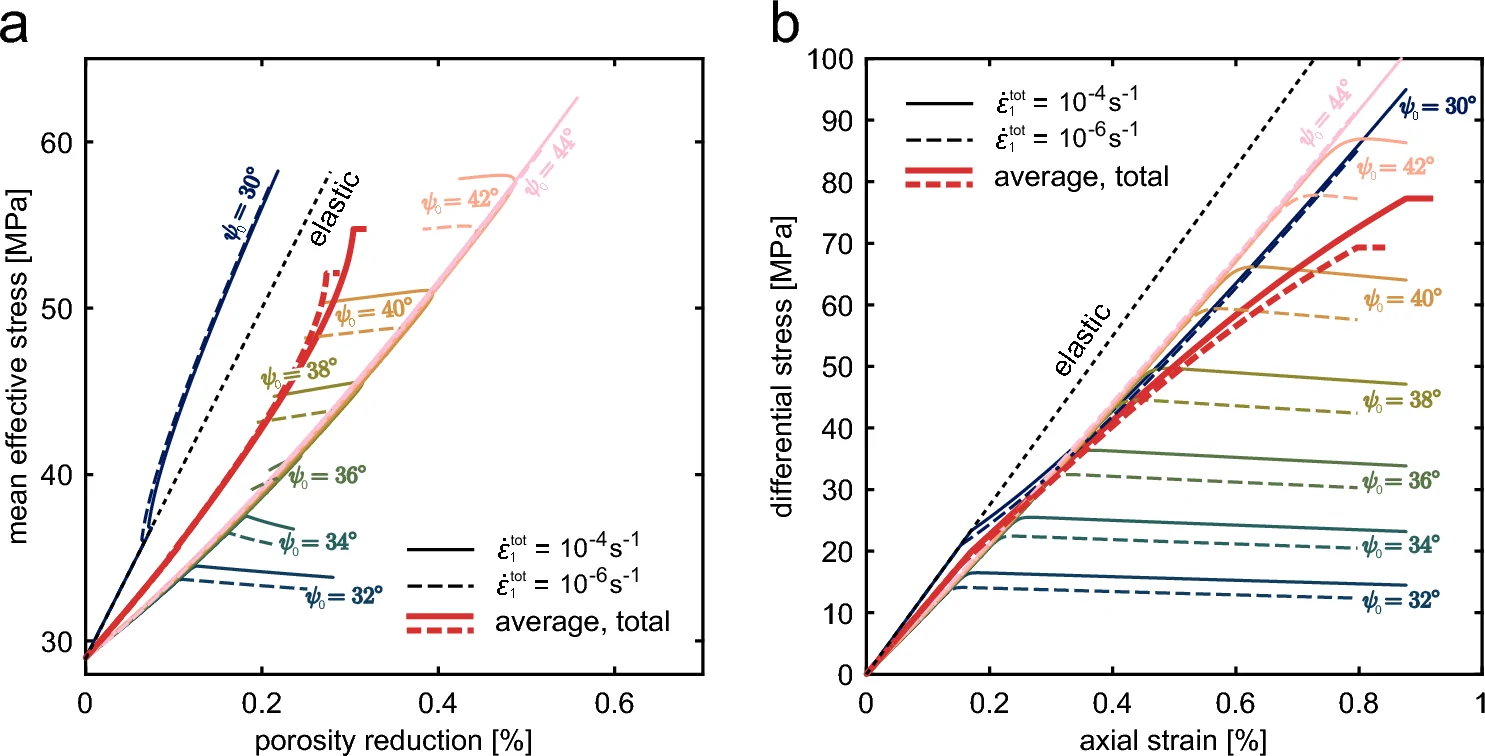
Modelled mechanical behaviours of unit cells with varying values of ${\psi }_{0}$ from ${30}^{\circ }$ to ${44}^{\circ }$, used to yield the integrated mechanical behaviours (red curves) at axial strain rates of ${10}^{-4}$ and ${10}^{-6} \, {\mathrm{s}}^{-1}$ presented in Fig. 5. **a** Mean effective stress P versus total porosity reduction $\Delta {\phi }_{\mathrm{tot}}$, and **b** differential stress Q versus total axial strain ${\varepsilon }_{1}^{\mathrm{tot}}$. Note that strain rate varied per model run, as indicated

The modelled mechanical behaviour for unit cells with $\frac{\pi }{6}\le {\psi }_{0}\le \frac{\pi }{4}$ ($30^\circ \le {\psi }_{0}\le 45^\circ$), at axial strain rates on the order of ${10}^{-4}$ and ${10}^{-6} \, {\mathrm{s}}^{-1}$ (Fig. 6), are shown in Fig. 6. For illustrative purposes, the mechanical behaviour is presented at every ${2}^{\circ }$ interval in ${\psi }_{0}$, from ${\psi }_{0}={30}^{\circ }$ to ${44}^{\circ }$.

For cells with ${\psi }_{0}>{30}^{\circ }$, the model results show near-identical, quasi-linear stress vs. porosity reduction behaviour in all unit cells, regardless of the values of ${\psi }_{0}$ (Fig. 6a). This behaviour is dominantly caused by intergranular clay film compaction, in addition to elastic deformation (see excess porosity reduction, compared to elastic curve shown with dashed line; Fig. 6a). It is followed by strain softening behaviour, characterized by net compaction in unit cells with ${30}^{\circ }<{\psi }_{0}\le {35.3}^{\circ }$ or net dilation in unit cells with ${35.3}^{\circ }<{\psi }_{0}<45^\circ .$ This softening behaviour is caused by the unloading effect of intergranular slip, occurring when axial strain rate ${\dot{\varepsilon }}_{1}^{\mathrm{slip}}$ due to slip becomes faster than the imposed axial strain rate ${\dot{\varepsilon }}_{1}^{\mathrm{tot}}$. This intergranular slip is accompanied by a decrease in ψ (cf. Eq. 18), which becomes more significant with decreasing ${\psi }_{0}$ such that the total decrease in ψ in unit cells with ${\psi }_{0}={44}^{\circ }$ and ${\psi }_{0}={31}^{\circ }$ are ${0}^{\circ }$ and ${0.53}^{\circ }$, respectively. Weakening was observed with decreasing axial strain rate in all unit cells, whether dilating or compacting (Fig. 6a). This rate effect was also caused by intergranular slip, with the magnitude of the effect being more pronounced in unit cells with higher ${\psi }_{0}$ (see the increased gap between the stresses supported at strain rates of ${10}^{-4}$ and ${10}^{-6} \, {\mathrm{s}}^{-1}$, with increasing ${\psi }_{0}$). Note that this difference in volumetric behaviour (i.e., compaction vs. dilation in Fig. 6a) is attributable solely to the geometry of the unit cells, whose porosities ϕ ($=1-\pi /\left[12{R}_{\mathrm{sc}}^{3}{\mathrm{sin}}^{2}\psi \mathrm{cos}\psi \right]$) are largest when $\psi ={35.3}^{\circ }$ and decrease as ψ deviates from this value, meaning that grain boundary sliding, accompanying reduction in ψ (cf. Eq. 18), leads to porosity reduction when ${\psi }_{0}<{35.3}^{\circ }$ and porosity increase when ${\psi }_{0}>{35.3}^{\circ }$.

By contrast, for cells with ${\psi }_{0}={30}^{\circ }$, the model calculations show quasi-linear behaviour throughout deformation (Fig. 6a). The onset of inelastic deformation occurred at a mean stress of 36–$37 \, \mathrm{MPa}$, resulting in slight dilation, that is reduced net compaction, compared to elastic behaviour (see reduced porosity reduction, compared to elastic curve shown with dashed line; Fig. 6a). A minor to modest rate effect was observed in this unit cell, since rate-independent clay consolidation prevents the rate of grain boundary sliding from increasing (i.e., clay consolidation is the rate-controlling process).

Having examined the mechanical behaviour of individual unit cells, it is now evident that the upward turn, observed in the integrated stress vs. porosity reduction behaviour (i.e., the behaviour of the reference case mix of cells) at P=43–$47 \, \mathrm{MPa}$  (shown in red; Fig. 6a), is attributable to the dilatational behaviour seen in the unit cells with ${\psi }_{0}>{35.3}^{\circ }$ (Fig. 6a). Considering that an identical total axial strain rate is imposed on each unit cell, the variation observed in the volumetric behaviour of cells with different ${\psi }_{0}$ indicates that lateral strain is localized and varies with the value of ψ developed. This localization is caused by grain boundaries being more susceptible to sliding with increasing grain contact angle, which corresponds to a decreasing ψ-value. Likewise, axial stress is significantly heterogeneous or localized, in such a way that differential stresses supported at an axial strain of 0.8%, and at an axial strain rate of ${10}^{-4} \, {\mathrm{s}}^{-1}$, lie in the range 15–92 MPa (Fig. 6b).

### Parametric Analysis

**Fig. 7:**
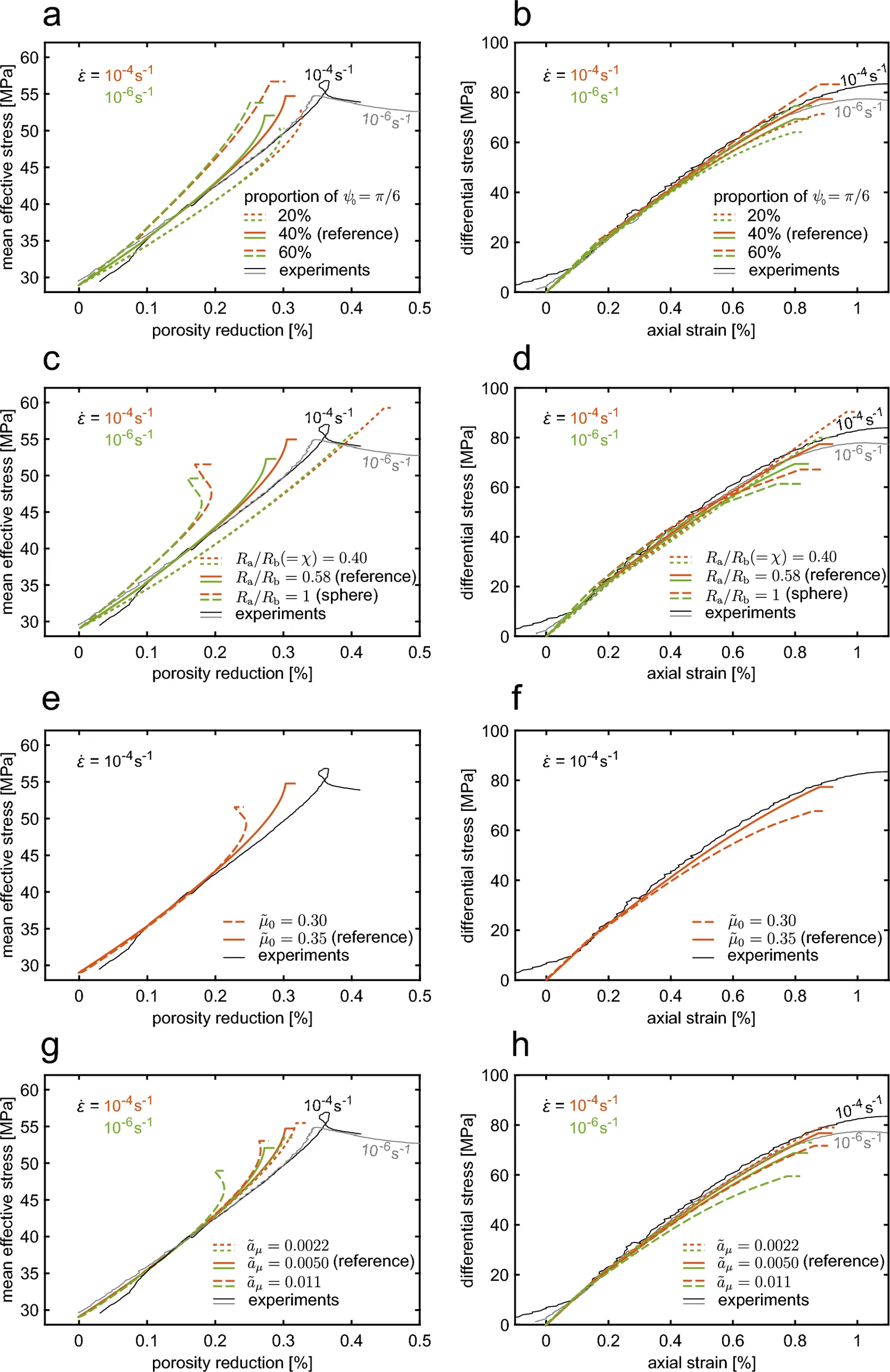

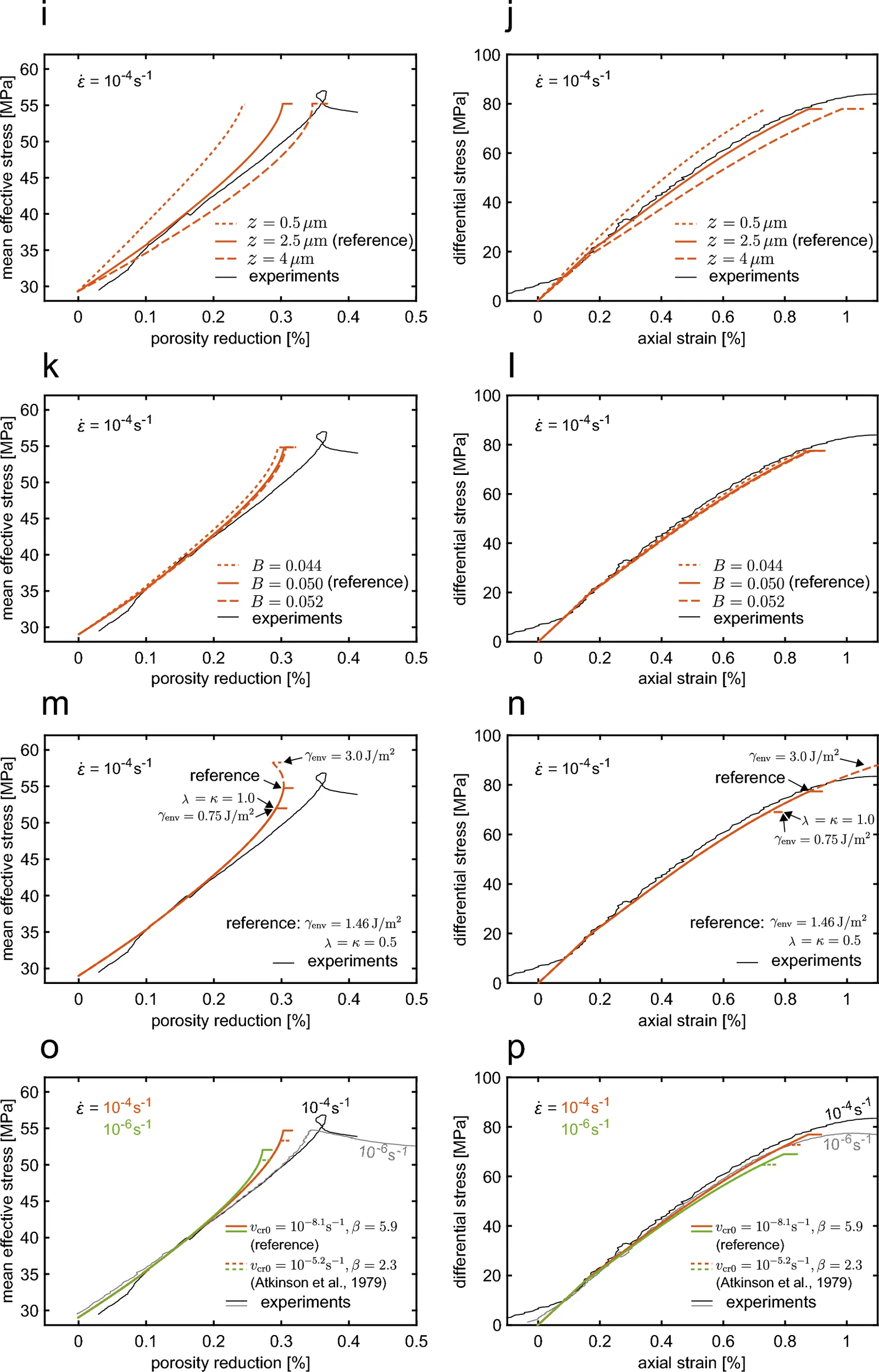
Sensitivity of modelled mechanical behaviour to variation in parameter values (the proportion of unit cells having ${\psi }_{0}=\frac{\pi }{6}$, $\chi ={R}_{a}/{R}_{b}$*, *${\widetilde{\mu }}_{0}$*, *${\widetilde{a}}_{\mu }$*, *z*, *B*, *${\gamma }_{\mathrm{env}}$*, *λ*, *κ*,* ${v}_{\mathrm{cr0}}$ and β*)*

To gain insight into the properties of the model, we performed a parametric analysis to investigate the sensitivity of the computed behaviour to the assumptions made and parameters used (i.e., to the proportion of unit cells with ${\psi }_{0}=\frac{\pi }{6}$ assumed and to the aspect ratio of grains $\chi ={R}_{\mathrm{a}}/{R}_{\mathrm{b}}$, the friction coefficient ${\widetilde{\mu }}_{0}$, measured at grain contact scale at reference velocity ${v}_{0}$, the rate-dependence coefficient in RSF terminology ${\widetilde{a}}_{\mu }$, intergranular clay thickness z, clay compaction parameter B, specific interfacial energy of fluid-filled cracks within quartz/clastic grains ${\gamma }_{\mathrm{env}}$, geometrical constants describing the meridional cracking λ and κ, and crack velocity parameters ${v}_{\mathrm{cr}0}$ and β; Fig. 7). To do so, we varied these parameters in realistic ranges about the values used in the reference case model. The analysis shows that the P vs. $\Delta {\phi }_{\mathrm{tot}}$ and the Q vs. ${\varepsilon }_{1}^{\mathrm{tot}}$ curves obtained exhibit a strong dependence on the proportion of unit cells with ${\psi }_{0}=\frac{\pi }{6}$ (Fig. 7a and b), the aspect ratio of grains ($\chi ={R}_{\mathrm{a}}/{R}_{\mathrm{b}}$, Fig. 7c and d), the friction coefficient at the grain contact scale at the reference velocity (${\widetilde{\mu }}_{0}$, Fig. 7e and f), the rate-dependence coefficient in RSF terminology (${\widetilde{a}}_{\mu }$, Fig. 7g and h) and the intergranular clay thickness (z, Fig. 7i and j). By contrast, little to no dependence of the overall stress–strain behaviour was observed on the clay compaction parameter (B, Fig. 7k and l), the specific interfacial energy of fluid-filled cracks within quartz grains (${\gamma }_{\mathrm{env}}$, Fig. 7m and n), the geometrical parameters for meridional cracking (λ and κ, Fig. 7m and n) and the crack velocity parameters (${v}_{\mathrm{cr}0}$ and β, Fig. 7o and p).

Increasing the proportion of unit cells having ${\psi }_{0}=\frac{\pi }{6}$ compared with the reference case of 40% (while keeping a fixed distribution in the range $\frac{\pi }{6}<{\psi }_{0}<\frac{\pi }{4})$, or decreasing clay film thickness z produced similar effects on the shapes of the P vs. $\Delta {\phi }_{\mathrm{tot}}$ and the Q vs. ${\varepsilon }_{1}^{\mathrm{tot}}$ curves (compare Fig. 7a and i and Fig. 7b and j). Similarly, peak stress is dependent on the proportion of unit cells with ${\psi }_{0}=\frac{\pi }{6}$ (Fig. 7a and b), the aspect ratio of grains (${R}_{\mathrm{a}}/{R}_{\mathrm{b}}$, Fig. 7c and d), the friction coefficient at grain contact scale at the chosen reference velocity (${\widetilde{\mu }}_{0}$, Fig. 7e and f), the rate sensitivity parameter for frictional sliding (${\widetilde{a}}_{\mu }$, Fig. 7g and h), the specific interfacial energy (${\gamma }_{\mathrm{env}}$, Fig. 7m and n) and the geometrical parameters for meridional cracking (λ and κ, Fig. 7m and n). This means that, although a good fit, or even one of the best fits, is obtained employing the reference case parameters and assumptions, good agreement between modelled and measured behaviour (e.g., shape of the P vs. $\Delta {\phi }_{\mathrm{tot}}$ and the Q vs. ${\varepsilon }_{1}^{\mathrm{tot}}$ curves and peak stress) can be obtained by choosing a different combination(s) of values for these parameters. We note, however, that manipulating the various parameters to produce a good fit has little value other than to demonstrate that the model offers a viable (but not necessarily unique) explanation for the rate- or time-dependent deformation behaviour observed in experiments on Bleurswiller sandstone.

Interestingly, among all the parameters employed, the aspect ratio of grains ${R}_{\mathrm{a}}/{R}_{\mathrm{b}}$ showed a strong role in controlling mechanical behaviour, in particular the P vs. $\Delta {\phi }_{\mathrm{tot}}$ behaviour (Fig. 7c). Good agreement between modelled and experimental data are not obtained unless ${R}_{\mathrm{a}}/{R}_{\mathrm{b}}$ is <1 (i.e., grains need to be flattened). This implies that grain shape must be taken into account when modelling the mechanical behaviour of sandstones, in addition to other parameters describing the deformation mechanisms, which in this case are frictional sliding, intergranular clay film compaction and stress corrosion cracking. This agrees well with previous DEM studies that showed grain shape controls the onset and evolution of shear banding in sands (e.g., Kawamoto et al. 2018).

## Model Evaluation, Suggestions for Future Improvement and Implications

We have shown that the main experimental trend in Q-P-ε-$\Delta \phi$ behaviour observed in experiments on Bleurswiller sandstones (Shinohara et al. 2024) can be accounted for by the present model. However, discrepancies were also found, including the non-smooth Q-P-ε-$\Delta \phi$ behaviour at the strain increment caused by stress corrosion cracking, which was not observed in experiments. This discrepancy mainly results from the simplifications underlying our assumptions of a regular array of identical ellipsoidal grains and their contact geometry at given ${\psi }_{0}$, plus the assumed identical clay films. These microscale features and properties will be distributed in natural sandstones, so that the Q-P-ε-$\Delta \phi$ behaviour is likely to be smoothed out. Such microstructural parameter variations and distributions need to be accounted for in the framework of a more rigorous grain-scale to aggregate-scale averaging approach to better quantify the mechanical behaviour exhibited by Bleurswiller sandstone and other porous sandstones. This challenging task can be approached in future using classical homogenization treatments (Bardet and Vardoulakis 2001; Fortin et al. 2003), thermodynamically based granular mechanics modelling methods (Einav 2007a, 2007b; Tengattini et al. 2014) and the discrete element method (Kawamoto et al. 2018).

The present microphysical model contributes to understanding of the processes governing time-dependent inelastic deformation, particularly at the small stresses and strains relevant for reservoir compaction during and after gas production. The model can thus be used to underpin the applicability of existing geomechanical models for hydrocarbon production-induced subsidence and seismicity, at field conditions and timescales (e.g., Aben et al. 2025; Pruiksma et al. 2015). An important finding is that the aspect ratio of grains, in addition to the presence of intergranular clay films (i.e., determining rate-dependent frictional parameters), is a key factor in determining the mechanical behaviour of porous sandstone at low stresses and strains, relevant for reservoir compaction during and after gas production. In principle, those parameters can be quantified using microstructural analysis methods such as X-ray micro-computed tomography (micro-CT) and scanning electron microscopy; hence, the time-dependent deformation behaviour of sandstone could in the future be quantified on the basis of digital microstructural and image analysis.

## Conclusions

In this study, a series of simplified microphysical models has been derived to explain the time-dependent inelastic deformation behaviour observed in triaxial compression experiments performed on a porous, clay-bearing sandstone, the Bleurswiller sandstone, under in situ conditions of temperature ($100 \, ^{\circ}{\rm C}$), pore water pressure and stress corresponding to the seismogenic centre of the Groningen gas field (Shinohara et al. 2024). In particular, we sought a mechanistic basis for the observed time-dependent inelastic deformation seen at the small strains (<1%) relevant to compaction of the reservoir in the Groningen gas field. On the basis of microstructural evidence obtained in previous experiments, inelastic deformation was inferred to be governed by time-independent consolidation of intergranular clay films, time-dependent intergranular slip and time-dependent intragranular splitting caused by stress corrosion mechanism. To illustrate variable grain packing geometries observed in the microstructures, our simplified sandstone model consists of a mixture of two unit cell microstructures, one having horizontal and inclined grain contacts and the other only having inclined contacts, with an assumed proportion and distribution of each unit cell. Both of these cell microstructures were taken to consist of a regularly packed 3D array of ellipsoidal quartz/clastic grains (long axis ${R}_{\mathrm{b}}=110$ μm, short axis ${R}_{\mathrm{a}}=64$ μm) having their short axis aligned parallel to the axial loading direction and having flattened contacts. Half of the unit cells contain intergranular clay films (thickness 2.5 μm) in all the flattened contacts, and the other half misses clay films in 50% of the inclined grain contacts (i.e., inclined grain contacts A not intersecting with the basal plane; cf. Fig. 3b) so that no traction is present. The model behaviour was explored for the stress conditions employed in the experiments in Shinohara et al. (2024), i.e., a confining pressure of $29 \, \mathrm{MPa}$ and differential stresses up to $85 \, \mathrm{MPa}$. This allowed a direct comparison between the modelled and experimentally obtained behaviour. We concluded the following points:

1. Our model captures the main trends in P versus total ($\Delta {\phi }_{\mathrm{tot}}$) and inelastic ($\Delta {\phi }_{\mathrm{inel}}$) porosity reduction and in stress–strain behaviour observed in the experiments on Bleurswiller sandstone.
2. The rate effect observed in the experiments and model at Q-values below the peak stress is caused by intergranular slip along clay films, which results in either compaction or dilation depending on grain contact angle. The difference in strain (i.e., strain localization) is accompanied by significant axial stress heterogeneity or localization, such that, at an axial strain of 0.8% and at an axial strain rate of ${10}^{-4} \, {\mathrm{s}}^{-1}$, differential stresses locally range from 15 to 92 MPa, while the average, sample-scale differential stress is 73 MPa.
3. We infer that the processes assumed in the present, simplified model, i.e., rate-independent clay consolidation, rate-dependent intergranular slip and intragranular cracking caused by stress corrosion mechanism, can indeed account for the main trends in inelastic deformation behaviour seen in our experiments. The discrepancies between the model predictions and the observations mainly result from the simplifications underlying in our assumption of a regular array of identical ellipsoidal quartz/clastic grains and contact geometry at a given ${\psi }_{0}$ and identical clay films. This is in reality likely to be smoothed out due to distributed values of grain (contact) geometries.
4. The present model provides mechanistic underpinning for existing geomechanical models accounting for rate-dependent deformation relevant to hydrocarbon production-induced subsidence and seismicity, at field conditions and timescales.
5. In future, the mechanical behaviour exhibited by Bleurswiller sandstone and other porous sandstones with intergranular clay films and relatively homogeneous porosity, should be better quantifiable by incorporating more rigorous grain- to aggregate-scale averaging procedures. This can be approached using methods such as classical homogenization treatments, thermodynamically based granular mechanics modelling methods and the discrete element method.

## Data Availability

The MATLAB code used to calculate the integrated stress versus elastic and inelastic strain behaviour is available via https://doi.org/10.24416/UU01-2PIB24. Yoda data publication platform of Utrecht University.
